# Harnessing discrete choice experiments to elicit preferred configurations of trustworthy AI augmented decision support systems for certified crop advisors

**DOI:** 10.3389/frai.2026.1747663

**Published:** 2026-05-13

**Authors:** Asim Zia, Maaz Gardezi, Xinjing Yu, Benjamin Ryan, Scott Merrill, Eric Clark, Ali Dadkhah, Donna M. Rizzo, John McMaine, David Clay

**Affiliations:** 1 Department of Community Development and Applied Economics, University of Vermont, Burlington, VT, United States; 2 Department of Computer Science, University of Vermont, Burlington, VT, United States, Burlington, VA, United States; 3 Urban and Environmental Policy and Planning, School of Public and International Affairs, Virginia Tech, Blacksburg, VA, United States; 4 Department of Community Development and Applied Economics, University of Vermont, Burlington, VT, United States; 5 Department of Agriculture, Landscapes and Environment, University of Vermont, Burlington, VT, United States; 6 Department of Civil and Environmental Engineering, University of Vermont, Burlington, VT, United States; 7 Department of Biosystems and Agricultural Engineering, University of Kentucky, Lexington, KY, United States; 8 Department of Agronomy, Horticulture and Plant Science, South Dakota State University, Brookings, SD, United States

**Keywords:** AI perceptions, choice experiments, decision support systems, precision agriculture, trustworthy AI

## Abstract

**Introduction:**

The increase in Artificial Intelligence (AI) and sensor data driven Precision Agriculture (PA) technologies show promise to improve efficiencies in agricultural production systems and decrease adverse impacts of agriculture on environment compared to traditional approaches. Yet, complex trade-offs (e.g. cost, accuracy, precision and data ownership) in the design and configuration of trustworthy AI augmented decision support systems (AI-DSS) for advancing responsible and ethical PA have surfaced. This study harnesses Discrete Choice Experiments (DCEs) to elicit stated preferences of Certified Crop Advisors (CCAs) for informing the design and configurations of trustworthy AI-DSS. The research is guided by two questions and eight associated hypotheses: (a) How do cost, accuracy, precision, and data ownership influence the preferences of CCAs for adopting AI-DSS in agriculture? (b) Which AI perceptions, PA technology concerns and prior DSS experience predict the adoption of AI-DSS configurations?.

**Methods:**

Six focus groups informed the design of the choice set, comparing low, medium and high cost AI-DSS with varying accuracy, precision and data ownership attributes. The survey was circulated by Crop Science Society of America to ~2600 CCAs with a lottery-based incentive, leading to 771 responses (response rate = 29.65%). The DCE data were analyzed using a Standard (McFadden) Logit Model, and a Random Utility Mixed Logit Model.

**Results:**

Analysis showed 25.54% of the participants opted out, and 45.36%, 19.23%, 9.85% prefer low, medium and high-cost AI-DSS, respectively. Marginal improvement of 1% accuracy leads to ~4% (*p* < 0.001) and spatial precision leads to ~ 1.5% (*p* < 0.01) increment in the likelihood of AI-DSS adoption.

**Discussion:**

AI perceptions, PA technology concerns and prior DSS experience significantly predict the variability in the adoption of three types of AI-DSS.

## Highlights

- Techno-optimism drives preference for advanced AI-DSS among crop advisors
- High cost significantly reduces adoption of intermediate and advanced AI-DSS
- Baseline interest exists as even basic AI-DSS is preferred over opting out entirely
- Trust and usability concerns strongly deter selection of complex AI-DSS tools
- Privacy concerns lower preference for basic AI-DSS, likely due to perceived weak security

## 1 Introduction

The increasing integration of artificial intelligence (AI) into agriculture is changing the way farm management decisions are made. A central development in this transformation is the emergence of AI-based decision support systems, or AI-DSS. These tools utilize highly detailed data collected through satellites, drones, and soil-based sensors, and apply AI algorithms on these multi-scaled data to provide more accurate and precise recommendations to farmers in real time and across specific locations. The emergence of AI-DSS builds of the longstanding efforts to integrate Decision Support Systems (DSS) with precision agriculture (PA), which have historically consisted of a suite of data-intensive tools that help farmers make timely and targeted decisions across crop, livestock, and environmental management (Finger et al., 2019; Lioutas et al., 2019; Viscarra Rossel and Bouma, 2016).

Decision support systems (DSS) within PA are not new. Their earliest forms were developed in the 1970s with advances in electronics and computing (McCown et al., 2002; Zhai et al., 2020). However, recent improvements in digital technologies have significantly expanded their capabilities. Today, DSSs combine AI, cloud computing, satellite-based remote sensing, and big data analytics to generate highly specific and actionable guidance for farmers (Kamilaris et al., 2017). These tools now deliver recommendations on when and how to plant, fertilize, irrigate, and manage pests, contributing not only to farm profitability but also to broader environmental goals such as soil health, water conservation, and climate resilience (Hannah et al., 2020; Jung et al., 2021; Khanna, 2021; Lindblom et al., 2017; Mackrell et al., 2009; Sood et al., 2022).

A major distinction between different types of DSS lies in their underlying design and technology. Traditional process-based models, typically developed by universities and public institutions, rely on biophysical principles to simulate farm processes. These models are more transparent but are sometimes limited by low resolution or infrequent calibration (Liu et al., 2024). In contrast, many modern AI-DSS are developed by private companies and use large datasets to produce high-resolution, predictive outputs. These commercial systems, such as (Cropin Intelligence, 2026; Planet Insights Platform, 2026; Pléiades Neo for Agriculture, 2026), often outperform traditional systems in terms of accuracy and precision, but they are also more costly and raise concerns about access, fairness, and transparency (Gardezi et al., 2024a).

Despite these technical advances, adoption of AI-DSS in agriculture remains limited. Financial barriers are a major challenge. Larger farms are more likely to adopt these technologies due to greater access to capital and human resources (Khanna, 2021; Pignatti et al., 2015). Smaller farms, in contrast, face limited resources, higher relative costs, and lack of training opportunities that constrain adoption (Hall and Matos, 2010; Isgin et al., 2008; Kendall et al., 2022). Long return-on-investment timelines, maintenance costs, and uncertainty regarding economic benefits all add to farmers’ and crop advisors’ reluctance to adopt AI-DSS (Jakku and Thorburn, 2010; Lee et al., 2021).

The adoption of AI-DSS also depends heavily on trust in the technology and the organization providing the product and service. Farmers and advisors prefer systems that are not only accurate and precise, but also understandable, transparent, and secure (Thompson et al., 2019). As Chamola et al. (2023) note, the development of trustworthy and explainable AI requires more than technical robustness, it necessitates socially embedded design choices that align with users’ values, needs, and expectations. Trust in AI is shaped by multiple dimensions: the perceived accuracy and precision of the technology, its cost-effectiveness, the explainability and accountability of its outputs, and how user data are governed. In agricultural contexts, these trust factors are especially important. The sector involves diverse actors, highly variable farm environments, and substantial economic and environmental risk. Despite the emergence of high-performing AI models in agriculture, concerns about data privacy, model opacity, and unequal access continue to surface (Adereti et al., 2024; Brugler et al., 2024; Gardezi et al., 2024b). Several studies have shown that users are more likely to trust AI tools that can clearly explain their recommendations and that align with the users’ level and type of expertise (Bayer et al., 2022; Kim et al., 2021; Zhang and Schwarz, 2012). As the complexity of AI models increases, particularly in deep learning, these systems often become less interpretable and harder to trust, especially for users without specialized training (Afroogh et al., 2024). When AI systems deviate from conventional practices or offer unfamiliar results, users may choose to rely on human advisors instead (Bhatti et al., 2021; Kovari, 2024).

Another critical issue is data governance. Many AI-DSS require users to share large volumes of sensitive farm data, including information on crop types, fertilizer use, yields, and field conditions. Publicly developed tools usually ensure that users retain control over their data, while private tools may assert ownership or restrict access (Adereti et al., 2024; Brugler et al., 2024). These practices raise concerns about privacy, fairness, and long-term control of agricultural knowledge (Floridi, 2019; Idowu et al., 2023; Zhang and Schwarz, 2012).

Against this background, this study investigates the attributes that contribute to trust and adoption of AI-DSS in agriculture. We focus specifically on Certified Crop Advisors, or CCAs, who serve as a vital link between technology developers and end users. The design of this experiment draws directly from stakeholder engagement activities, including six focus group discussions conducted in South Dakota and Vermont with farmers, extension specialists, nonprofit organizations, and technology developers. These sessions emphasized the importance of cost, accuracy, precision, and data ownership in shaping trust in AI-DSS. For example, participants in Vermont debated the trade-offs between freely available satellite data and proprietary high-resolution data. In South Dakota, concerns about corporate control over farm-generated data were more prominent. Across locations, stakeholders agreed that AI-DSS need to be not only high-performing but also user-centered, fair, and transparent.

This study aims at estimating the multi-attribute value functions of CCAs for cheaper, medium range and expensive AI-DSS with various accuracy, precision and data ownership structures. Using a Discrete Choice Experiment (DCE), we evaluate the willingness of CCAs to pay for different AI-DSS configurations. DCEs, supplemented with conjoint analysis, provide a well-established approach to estimate the CCA market demand for various configurations of AI-DSS, and infer the trust placed on purchasing an AI-DSS conditional upon its underlying attributes and levels of attributes.

Discrete Choice Experiments (DCEs) are increasingly valuable for evaluating trade-offs among different attributes of Decision Support Systems (DSS), particularly in complex, multi-criteria environments such as food-water-energy nexus, finance, and public policy (Shang and Chandra, 2023). DCEs allow researchers and stakeholders to systematically assess user preferences by presenting hypothetical scenarios where various system attributes—such as transparency, accuracy, response time, and user control—are varied. DCE’s elicit choices between alternatives, enabling the estimation of the relative importance of each attribute and the trade-offs users are willing to make. This method is especially useful in AI contexts where ethical, functional, and usability considerations must be balanced. For instance, DCEs can quantify the relative value of precision over predictive accuracy, or data sovereignty over corporate control of data. The DCE technique’s strength lies in its ability to simulate real-world decision-making while maintaining experimental control, making it a robust tool for informing the design and deployment of AI systems. The overall objective of a DCE is to estimate marginal and conditional values for multiple attributes of a good or service that is the subject of decision analysis. Including price as a characteristic permits a multidimensional, preference-based valuation topology to be estimated for use in the design of innovative technologies such as AI-DSS.

DCEs have gained popularity because they offer several potential advantages relative to other valuation methods (Holmes et al., 2017). DCEs can provide marginal and conditional values for changes within multiple levels of attributes as well as among multiple attributes, resulting in a response surface of multiple values rather than a single value. Because attributes and levels of attributes are experimentally manipulated and presented to respondents, they are typically exogenous, not collinear, and can reflect attribute levels outside the range of the current market or environment (Holmes et al., 2017). The stated preference DCE method has an advantage over revealed preference data that are often collinear, may be limited in variation, and may be endogenous in explaining choices (Holmes et al., 2017). DCEs are often used to assess preferences and trade-offs in behavioral settings, including AI-enabled decision support tools in agriculture, healthcare, transportation, and public policy (Vo et al., 2025; Zhu et al., 2024; Zhu et al., 2026; Ma et al., 2025; Ju et al., 2023; Rafaï et al., 2025; Khanna et al., 2024). The potential advantages of DCE’s are, however, limited by many challenges. Given that DCE responses are stated preferences, inference validity concerns are well known that arise due to the strategic behavior or hypothetical bias (Meginnis et al., 2021; Menapace and Raffaelli, 2020). The cognitive difficulty faced by respondents in considering alternatives with multiple attributes in new choice situations may also be high. Requiring respondents to assess complex trade-offs may result in behavioral responses such as the use of decision heuristics that are not well understood and might not reflect how they would make actual market choices (Holmes et al., 2017).

The research is guided by the following questions: (a) How do cost, accuracy, precision, and data ownership influence the preferences of Certified Crop Advisors for adopting AI-DSS in agriculture? (b) Which AI perceptions, PA technology concerns and prior DSS experience predict the adoption of AI-DSS configurations?

To predict the configural preferences of AI-DSS adoption by different users and advance theory of trustworthy AI (technology) design, we posited and tested the following hypotheses through the DCEs reported in this study:

*H1:* Crop advisors are generally more likely to choose some form of AI-DSS over opting out entirely, even if it is a lower-performing tool like AI-DSS 1, indicating broad baseline interest.

*H2:* Crop advisors who believe AI will transform future agricultural work are more likely to prefer higher-performing AI systems, including moderately advanced (AI-DSS 2) and cutting-edge (AI-DSS 3) options.

*H3:* Higher costs reduce the likelihood of selecting tools, particularly the most advanced (AI-DSS 3), reflecting economic constraints among potential users.

*H4:* Crop advisors already familiar with AI products may be more critical or selective, avoiding “in-between” systems like AI-DSS 2, in favor of either simpler (AI-DSS 1) or more sophisticated alternatives (AI-DSS 3).

*H5:* Lack of trust in AI recommendations significantly reduces preference for all DSS types, with the greatest sensitivity likely in advanced systems (AI-DSS 2 and 3) due to their complexity.

*H6:* Crop advisors who express usability concerns tend to prefer more basic systems like AI-DSS1, indicating that interface simplicity may outweigh model performance for these users.

*H7:* Crop advisors who enjoy experimenting with tools are much more likely to prefer advanced systems, especially AI-DSS 3, reflecting alignment with their desire to explore and understand.

*H8:* Data privacy concerns reduce preference for AI-DSS 1, potentially due to perceived lack of transparency or security in low-cost systems.

## 2 Methods

### 2.1 Experimental data collection procedures

**Figure 1:**
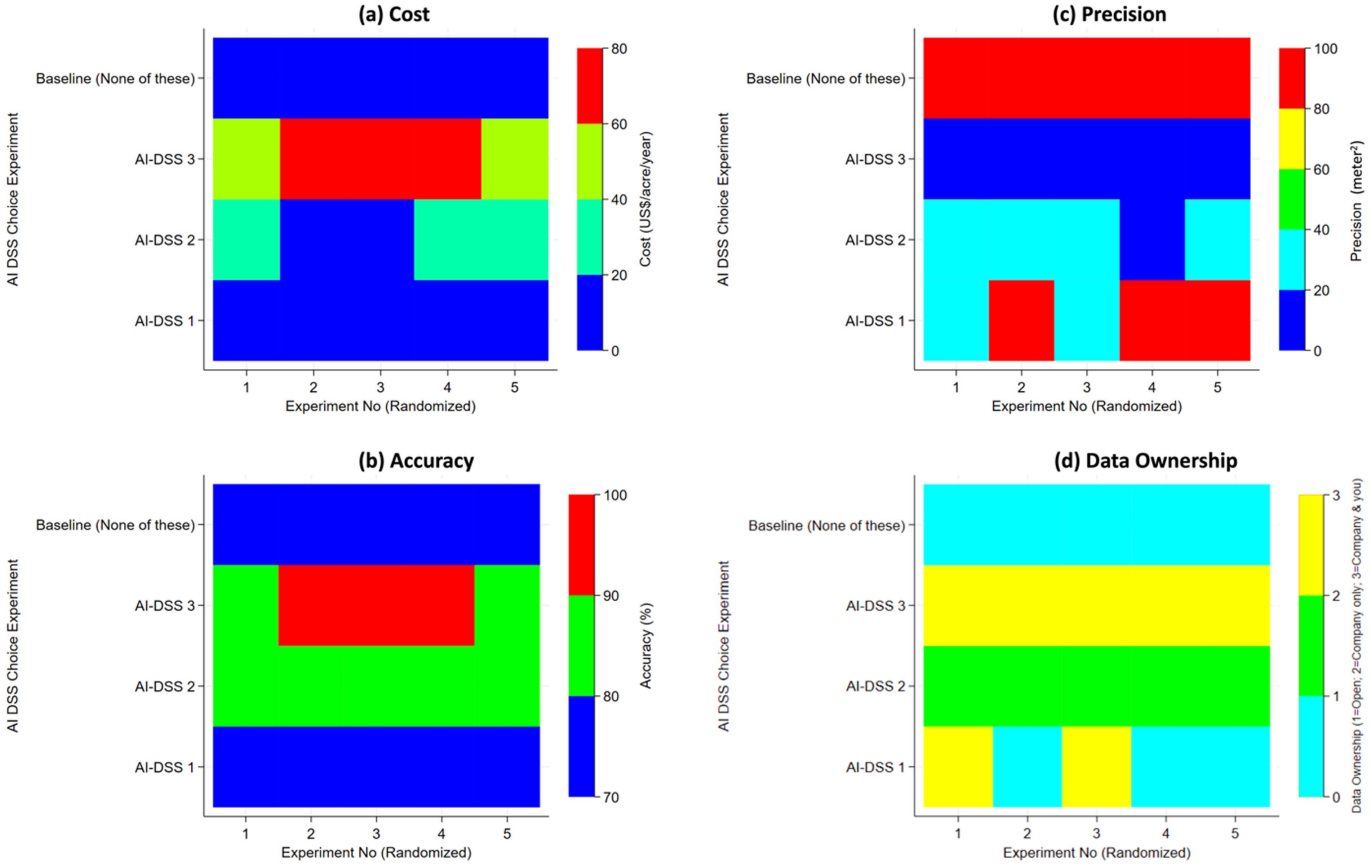
Heat map showing configural design of five randomized DCEs (x-axis) for three configurations of AI-DSS with opt out option (y-axis) by different levels of cost **(a)**, accuracy **(b)**, precision **(c)** and data ownership **(d)**.

Six focus groups informed the design of the DCE choice set, comparing low, medium and high cost AI-DSS with varying accuracy, precision and data ownership attributes (Table 1 and Figure 1). The survey was circulated by the Crop Science Society of America (CSSA) among its mailing list of ~2,600 CCAs within North America with a lottery-based incentive, leading to 771 responses (response rate = 29.65%). Under the ‘lottery-based’ incentive, 50 out of 771 respondents were randomly selected for an incentive payment of $20 per respondent. The DCE survey protocol, long-form version of survey response data in both STATA SE 19.5 and CSV format and associated STATA code to estimate choice models (Section 2.2) are available in Harvard Dataverse, a public data repository. For replicating this survey on CCAs, researchers will need to sign a contract with CSSA administrators. All authors of this manuscript contributed towards the design of focus group protocols, and subsequent DCE survey protocols derived from the analysis of focus group data. The University of Vermont’s Research Protections Office approved these protocols under study # UVM IRB 00001891. Virginia Institute of Technology approved these protocols under study # VT IRB 24–1,255. The reliability of the survey responses was ensured through analysis of respondent login IDs, their IP addresses and timestamp metadata. The survey data was collected precisely between February 29th and March 20th of 2024. The raw data was extracted as CSV, evaluated for reliability, and converted into long-form in STATA SE 19.5 for estimation of choice models.

**Table 1:** Discrete choice experiment design for the five randomized choice tasks, showing the attribute levels for cost, predictive accuracy, spatial precision, and data ownership for each AI-DSS alternative and the opt-out option.

Attribute	Experiment # (randomized)	AI-DSS 1	AI-DSS 2	AI-DSS 3	None of these
Cost ($/acre/year)	1	$2	$30	$60	$0
	2	$0	$10	$80	$0
	3	$4	$10	$80	$0
	4	$0	$30	$80	$0
	5	$0	$30	$60	$0
Accuracy (%)	1	70%	85%	90%	70%
	2	70%	85%	99%	70%
	3	80%	85%	99%	70%
	4	70%	85%	95%	70%
	5	70%	85%	90%	70%
Precision (meter^2^)	1	25	25	1	100
	2	100	25	1	100
	3	25	25	1	100
	4	100	9	0.25	100
	5	100	25	1	100
Data Ownership (1 = Open) (2 = AI-DSS Company only) (3 = AI-DSS Company and you)	1	3	2	3	1
	2	1	2	3	1
	3	3	2	3	1
	4	1	2	3	1
	5	1	2	3	1

The choice experiment was framed as follows:

“Imagine comparing between artificial intelligence decision support systems (AI-DSS) that can help you decide what and when to plant, how much to irrigate & fertilize, and when to harvest. In the next five questions, you will have a choice between three AI-DSS that all use artificial intelligence, but they differ in their cost, accuracy, precision, and data ownership attributes.”

Each respondent completed five sequential choice tasks. In each task, respondents were presented with four alternatives: three AI-enabled decision support systems (AI-DSS 1–3) with experimentally varied attributes, and an opt-out alternative (‘None of these’). Respondents were asked to select exactly one alternative per task. Attribute levels were randomized across tasks and alternatives, such that respondents evaluated multiple hypothetical system configurations while holding the overall framing constant. Each of the five questions tested five experimental treatments, as shown in Table 1 and x-axis of Figure 1, that were randomized in their order of appearance. Each of the five experiments tested variability on the levels of attributes within the broader technological feasibility and design parameters inferred from the analysis of focus group data to compare three types of AI-DSS. The levels of attributes for each of the three contrasting types of AI-DSS were derived from synthesis of the focus group data. While market prices of the currently available commercial AI-DSS (e.g., Cropin Intelligence, 2026, Planet Insights Platform, 2026, Pléiades Neo for Agriculture, 2026) are not transparently available, our choice experiment design was driven by the stakeholder input that constrained the emergent “hypothetical market” availability of AI-DSS by three broad types in the near future spanning low-, medium- and high-cost systems. The cost of the cheapest AI-DSS 1 ranges from $0 to $4 per acre. In Experiment #2, 4 and 5, AI-DSS1 costs $0 with 70% predictive accuracy rate, 100 meter^2^ spatial precision and open data ownership. For respondents opting out of choosing any AI-DSS, AI-DSS 1 attribute values in experiments 2, 4 and 5 provided an anchor for our experimental design. Respondents opting out could, if interested, harness open data and source codes for acquiring AI-DSS1 free of cost capable of generating 70% accurate forecasts at 100 meter^2^ spatial precision. With some marginal increments in the cost of AI-DSS1, i.e., Experiment #1 at $2 and Experiment #3 at $4, AI-DSS1 can be designed to improve predictive accuracy to 80% and spatial precision to 25 meter^2^. In contrast to AI-DSS1, the most expensive AI-DSS3 are the most advanced, complex and costly systems (ranging from $60 to $80 per acre per year) but these can attain predictive accuracy rates of 90 to 99% at cutting edge spatial precision (ranging from 0.25 to 1 meter^2^). The medium cost AI-DSS 2 (ranging from $10 to $30) attain 85% predictive accuracy at medium level spatial precision (9 to 25 meter^2^). One of the major determining factor in estimation of these cost-accuracy-precision triplets in designing the experiment was driven by our stakeholder-informed market analysis of current costs of the satellite data (Sentinel-1/2/3 free at 100 meter^2^ spatial precision) at the lower end and high costs of high resolution data (e.g., World-View and Hyperspectral satellite missions at 0.25 to 1 meter^2^ spatial precision) at the upper end. The medium cost AI-DSS2 represents Planet satellite system spatial precision at 9 to 25 meter^2^. Additional differentiating factors between the input costs of the three AI-DSS were based on stakeholder estimates of the cost of multi-spectral drones, *in situ* sensing systems and the marginally additional cost of developing and sustaining more advanced machine learning & AI algorithms that improve the predictive accuracy of target decision variables. To de-limit the number of experiments due to the constraint of survey respondent time, AI-DSS2 only tested for Company-only data ownership structure, while AI-DSS3 only tested for “Company and you” data ownership attribute levels. To ensure orthogonality of the experimental design, attribute levels for cost, accuracy, precision, and data ownership were independently varied across alternatives and tasks, subject only to technological feasibility constraints derived from focus group input. This design minimizes correlation among attributes and allows unbiased identification of marginal utilities. Figure 1 illustrates this orthogonality visually, showing that no attribute systematically covaries with another across experimental treatments. Aggregating predictive accuracy across decision domains (planting, irrigation, fertilization, harvesting) further preserved orthogonality while reducing cognitive burden on respondents. All tables and figures in this article refer to the choice of “opt out” as baseline (none of these) for theoretical consistency.

### 2.2 Experimental data analysis

**Figure 2:**
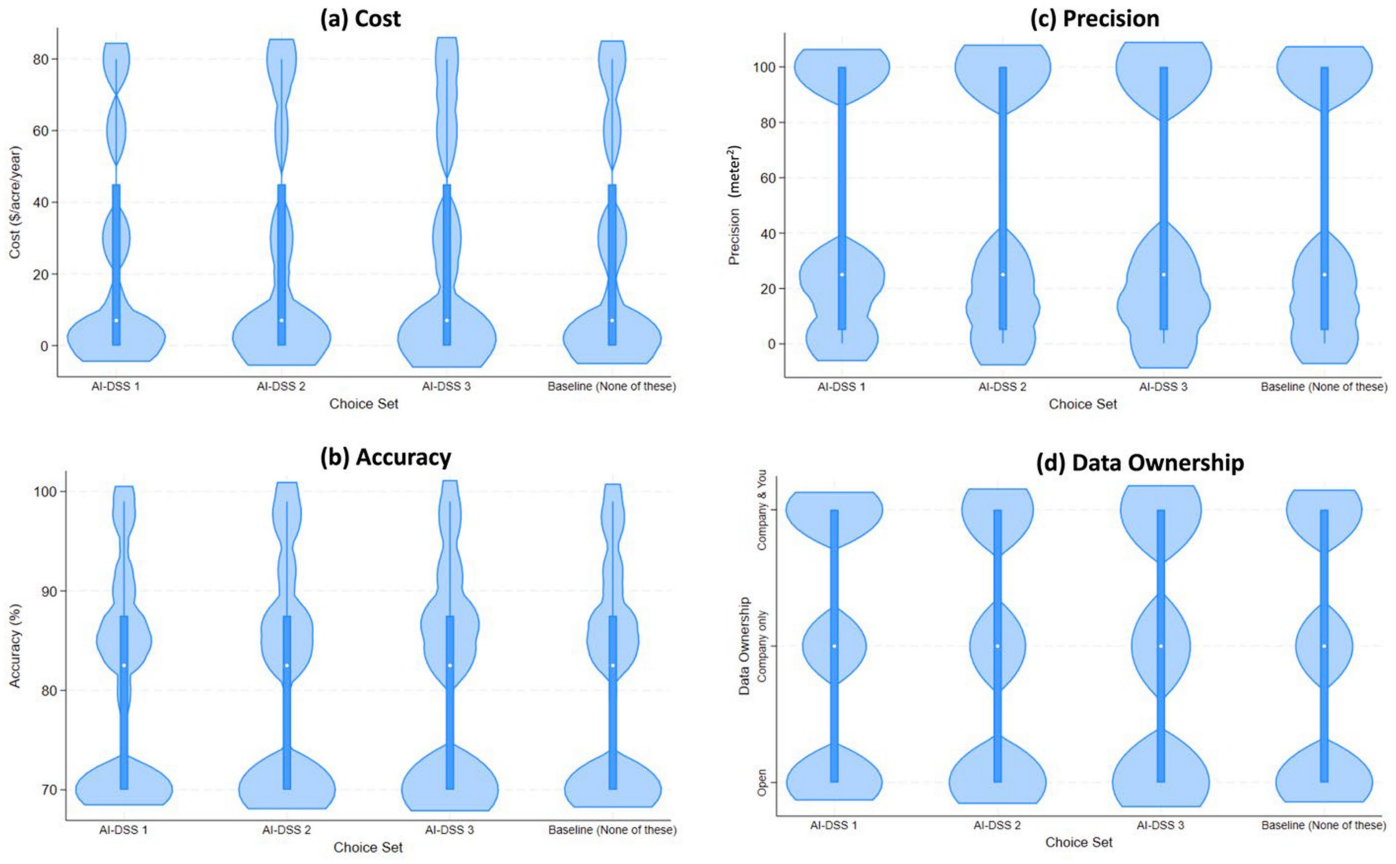
The violin plot shows AI-DSS choice set on x-axis and the median, range and density of cost **(a)**, accuracy **(b)**, precision **(c)** and data ownership **(d)**. In this violin plot, the open circle is a marker for the median, the thick line shows the interquartile range with whiskers extending to the upper and lower adjacent values. This is overlaid with a density of the data.

The choice experimental data were analyzed by using two alternate models: (a) Standard (McFadden) Logit Model; and (b) Random Utility Mixed Logit Model (Holmes et al., 2017). The DCE data set was converted from short to long form for estimating these two choice models. Each respondent who fully completed the survey provided five choice observations, one for each choice task. Because each task contained four alternatives, the choice dataset was converted into long form with one row per alternative per task per respondent. With 771 respondents, this yields a theoretical maximum of 5 × 4 × 771 = 15,420 observations. After accounting for nonresponse, the final analytical dataset contained 15,292 observations (Table 2). Figure 2 shows AI-DSS choice set on x-axis and the median, range and density of the cost, accuracy, precision and data ownership level depicted on the y-axis. Because each respondent provided multiple choices, observations are not statistically independent within individuals. To address this, we estimated both a standard McFadden logit model with respondent-clustered robust standard errors and a Random Utility Mixed Logit model that explicitly allows unobserved preference heterogeneity and correlation across repeated choices at the respondent level. This modeling strategy ensures consistent estimation and valid inference despite repeated observations per respondent (McFadden and Train, 2000; McFadden, 2001).

**Table 2:** Descriptive statistics derived from long form DCE data for choice attributes and respondent covariates capturing AI perceptions, PA concerns, DSS experience, and demographics used in the full model specifications.

Variable	DCE question	Observations	Mean	Standard deviation	Min	Max.
AI-DSS Choice	Which system would you prefer? (Please choose only one option)	15,292	0.25	0.4330	0	1
Accuracy (%)	DCE Table	15,292	80.4017	10.4145	70	99
Precision (meter^2^)	DCE Table	15,292	48.1494	43.2644	0.25	100
Data Ownership	DCE Table	15,292	1.9503	0.8646	1	3
Cost (US$)	DCE Table	15,292	23.7997	29.9959	0	80
AI perception items						
Good understanding of AI	I have a good understanding of what artificial intelligence is	14,272	3.6552	0.9351	1	5
AI changes work life	Agricultural products and services using artificial intelligence have profoundly changed my work life in the past 3–5 years	13,872	2.5438	1.0405	1	5
AI simplifies work	Agricultural products and services using artificial intelligence simplifies my professional work	13,612	2.9509	0.9226	1	5
AI benefits exceed risks	Agricultural products and services using artificial intelligence have more benefits than drawbacks	13,992	3.2115	0.8930	1	5
Awareness of AI products	I am aware of the types of agricultural products and services that use artificial intelligence	14,032	3.1564	1.0041	1	5
AI will change future work	Agricultural products and services using artificial intelligence will profoundly change my work life in the next 3–5 years	14,052	3.6697	0.9699	1	5
PA concern items						
High cost of equipment	The cost of purchasing and operating precision farming technologies is too high	14,252	2.6806	1.0423	0	4
Cost of maintenance	The cost of maintaining precision farming technologies is too high	14,272	2.5386	1.1301	0	4
Decisions take time	Interpreting and making decisions takes too much time	14,232	2.3130	0.9147	0	4
Usability concerns	Precision farming tools lack a user-friendly interface	14,252	2.5046	1.0974	0	4
Loss of farmer knowledge	Farmers’ knowledge will become less important as we rely more on information provided by precision farming technologies	14,272	1.9391	0.9215	0	4
Data use uncertainty	I am not sure I am using the data I collect as effectively as possible to advise farmers	14,212	2.5736	1.0948	0	4
Trust in recommendations	I am unsure whether to trust recommendations made by precision farming technologies	14,212	2.6501	0.9558	0	4
Validation concerns	I still need to field check the recommendations made by the precision farming technologies	14,272	3.3688	0.7256	0	4
Farm size concerns	Precision farming technologies are only beneficial for big farms	14,252	1.9595	0.8387	0	4
Lack of transparency	There is not enough clarity and transparency about data collection terms and conditions	14,252	2.8546	1.1646	0	4
Corporate misuse of power	I am concerned about data being harvested by corporations without consent	14,272	3.2662	0.8626	0	4
Data privacy concerns	I am concerned about risks of data privacy related to precision farming technologies	14,252	3.2481	0.8641	0	4
Corporate trust concern	I am concerned that corporations will use data for their benefit and not farmers’	14,252	3.1919	1.0208	0	4
Regulatory misuse concern	I am concerned that data from precision farming technologies could be used for regulatory purposes	14,212	3.1806	1.0083	0	4
DSS experience items						
Like engagement with DSS	I like to occupy myself in greater detail with Ag decision support systems	13,556	2.6119	0.6275	1	4
Like testing DSS functions	I like testing the functions of new Ag decision support systems	13,516	2.6919	0.6340	1	4
Use DSS out of obligation	I predominantly deal with Ag decision support systems because I have to.	13,536	2.3862	0.6274	1	4
Like intensive use of new DSS	When I have a new Ag decision support system in front of me, I try it out intensively	13,496	2.4573	0.6563	1	4
Enjoy learning new DSS	I enjoy spending time becoming acquainted with a new Ag decision support system	13,536	2.5348	0.6845	1	4
Only cares if DSS works	It is enough for me that an Ag decision support system works; I do not care how or why	13,516	2.0733	0.6807	1	4
Seeks detailed understanding of DSS	I try to understand how an Ag decision support system exactly works	13,536	2.8726	0.6418	1	4
Wants basic functional knowledge of DSS	It is enough for me to know the basic functions of an Ag decision support system	13,516	2.4732	0.6614	1	4
Aims to fully use DSS	I try to make full use of the capabilities of an Ag decision support system	13,516	2.7431	0.6346	1	4
Demographics						
Farm size by acres		10,860	5737.1340	21253.22	0	300,000
Experience by year		12,796	21.9799	79.1967	0	2000
Age group		13,376	3.8504	1.469	1	6
Gender (Male = 0, Female = 1)		13,036	0.1374	0.3443	0	1
Advanced Degree		13,436	0.2902	0.4539	0	1
Race		13,376	0.8609	0.3460	0	1
Self Employed		13,416	0.2510	0.4336	0	1
Employed by Ag establishment		13,416	0.1013	0.3018	0	1
Employed by Agribusiness		13,416	0.5038	0.5000	0	1

**Figure 3:**
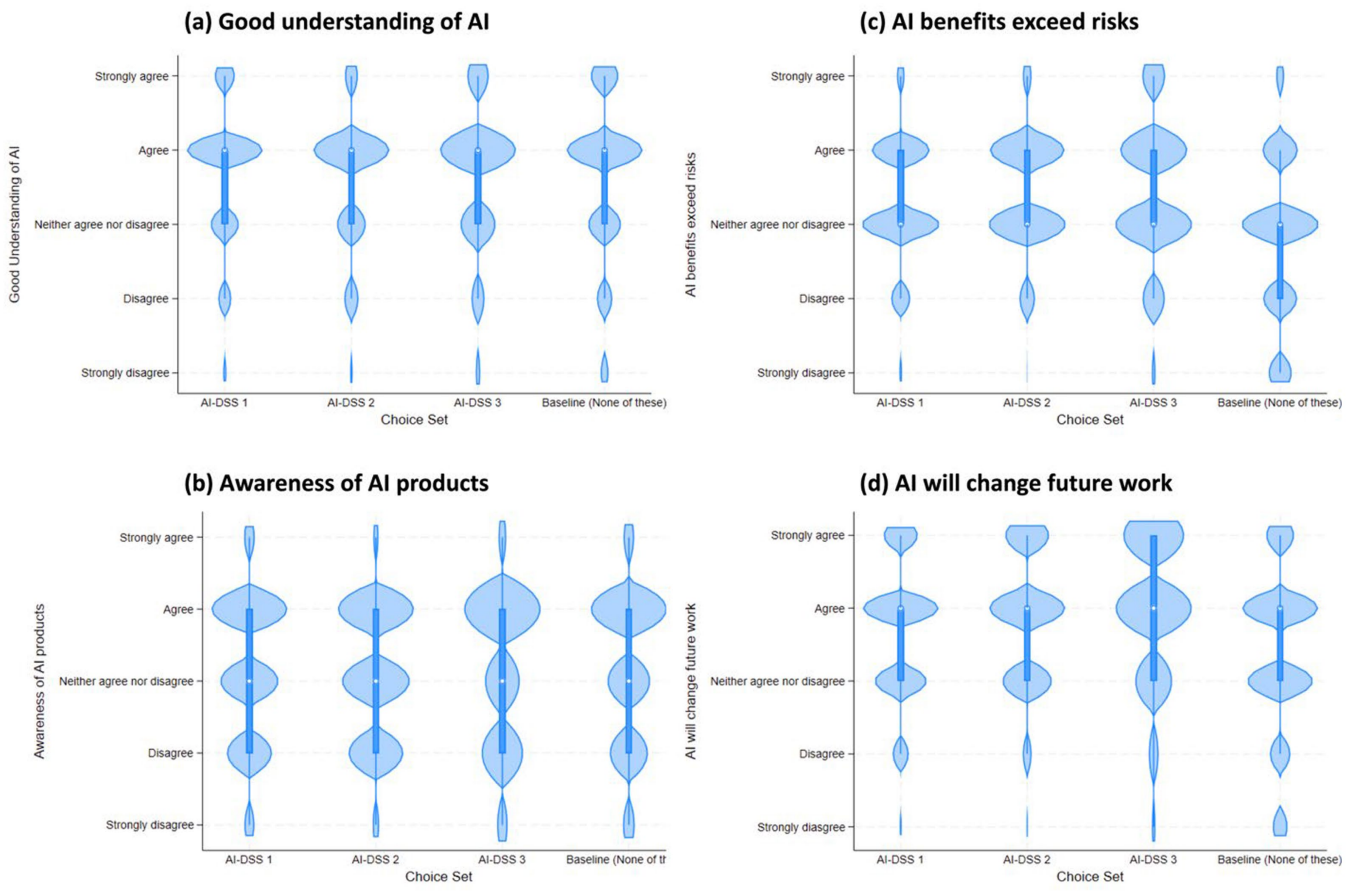
The violin plot shows AI-DSS choice set on x-axis and the median, range and density of four AI Perception survey items: **(a)** Good understanding of AI; **(b)** Awareness of AI products; **(c)** AI benefits exceed risks; and **(d)** AI will change future work. In this violin plot, the open circle is a marker for the median, the thick line shows the interquartile range with whiskers extending to the upper and lower adjacent values. This is overlaid with a density of the data.

In addition to the choice set level variables, the survey instrument included survey items to test four types of covariates: AI Perceptions, PA Concerns, DSS Experience and Demographics (See descriptives in Table 2). Six survey items asked respondents questions about their AI perceptions. Figure 3 shows the median, range and density of four of these AI perception items vis a vis the choices made by survey respondents.

**Figure 4:**
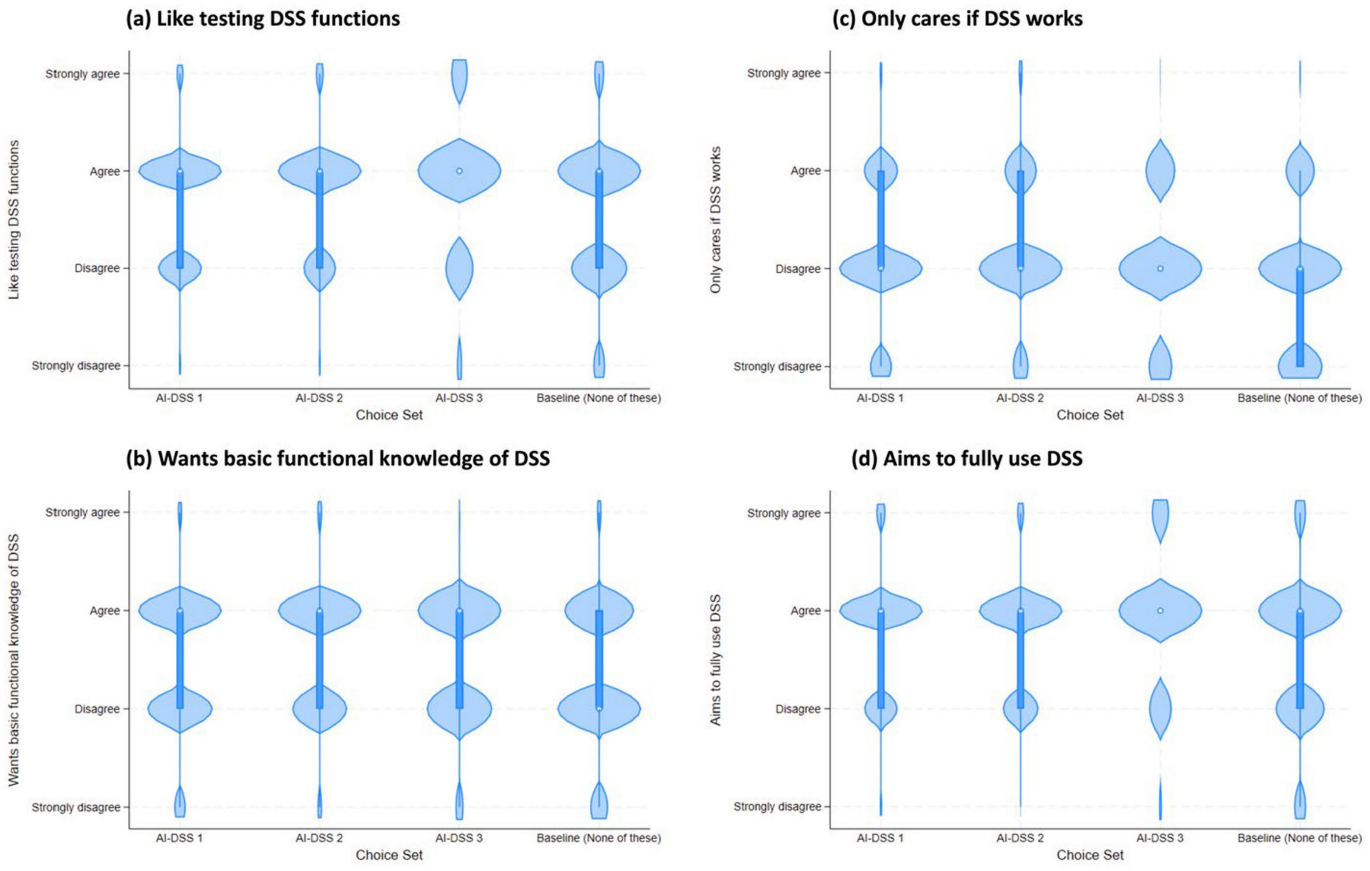
The violin plot shows AI-DSS choice set on x-axis and the median, range and density of four DSS experience survey items: **(a)** Like testing DSS functions; **(b)** Wants basic functional knowledge of DSS; **(c)** Only cares if DSS works; and **(d)** Aims to fully use DSS. In this violin plot, the open circle is a marker for the median, the thick line shows the interquartile range with whiskers extending to the upper and lower adjacent values. This is overlaid with a density of the data.

Fourteen survey items asked respondents questions about their PA concerns (Table 2). Further, nine survey items asked questions about their DSS experience. Figure 4 shows the median, range and density of four of these DSS experience items vis a vis the choices made by survey respondents. Finally, nine demographic questions collected information about the farm size of their operations, professional experience, age, gender, race, education and employment type. 98.72% of the survey respondents represented USA, while 0.89% represented Mexico; and 0.13% each self-identified to be from Great Britian, India and New Zealand. Country representation was tested but eventually not included in the choice models due to its insignificant effect.

STATA SE Version 19.5 was used to estimate two specifications of the Standard (McFadden) Logit Model and two specifications of the Random Utility Mixed Logit Model. Reduced models include a core set of alternative-specific utility attributes (cost, accuracy, precision, and data ownership). Full models further incorporate respondent-level covariates related to AI perceptions, PA concerns, DSS experience, and demographics, specified as alternative-specific effects for each AI-DSS alternative relative to opting out. Individual respondent-level variables (e.g., *good understanding of AI*) are represented by separate coefficients for AI-DSS 1, AI-DSS 2, and AI-DSS 3, tested individually with one degree of freedom per coefficient. In contrast, the Wald χ^2^ statistics reported in Table 3 reflect joint tests of parameter blocks defined by the model specification, rather than tests of individual coefficients. The reported degrees of freedom therefore correspond to the number of jointly tested parameter blocks (18 in the full models), not to the total number of alternative-specific coefficients displayed in the table. The model code and statistical output are made available in the Supplementary information for each of the four model results reported below.

**Table 3:** Estimated coefficients from choice experiments predicting respondents preferred configurations of AI-DSS.

Variable	M1: standard McFadden logit reduced model odds ratio (robust s.e.)	M2: standard McFadden logit full model odds ratio (robust s.e.)			M3: random utility mixed logit reduced model odds ratio (robust s.e.)	M4: random utility mixed logit full model odds ratio (robust s.e.)		
Choice set (alternative) specific variables								
Accuracy (%)	1.0316*** (0.0072)	1.0455*** (0.0109)			1.0301*** (0.0074)	1.0441*** (0.0112)		
Precision (meter^2^)	0.9922** (0.0043)	0.9887* (0.0057)			0.9896** (0.0047)	0.9856* (0.0073)		
Data Ownership	1.1092 (0.1917)	1.0282 (0.2406)			1.0208 (0.1873)	0.9341 (0.2526)		
Cost (US$)	0.9736** (0.0030)	0.9660*** (0.0041)			0.9700*** (0.0043)	0.9614*** (0.0083)		
Random Utility/ Normal sd(cost)					0.0123 (0.0059)	0.0142 (0.0127)		
ASC^+^ (for Opt Out alternative)	(base)	(base)			(base)	(base)		
ASC (AI-DSS1)	1.2381*** (0.0990)				1.235** (0.0987)			
ASC (AI-DSS2)	0.3992*** (0.0810)				0.3903*** (0.0803)			
ASC (AI-DSS3)	0.4303*** (0.0905)				0.4148*** (0.0893)			
Respondent (case) specific predictors								
		*AI-DSS 1*	*AI-DSS 2*	*AI-DSS 3*		*AI-DSS 1*	*AI-DSS 2*	*AI-DSS 3*
Good understanding of AI		1.1142 (0.1361)	0.9261 (0.1433)	1.1029 (0.2436)		1.1166 (0.1361)	0.9275 (0.1452)	1.0977 (0.2559)
AI changes work life		0.9066 (0.1146)	0.9157 (0.1406)	0.9656 (0.2060)		0.9065 (0.1148)	0.9139 (0.1422)	0.9800 (0.2226)
AI simplifies work		1.2746 * (0.1809)	1.0812 (0.1955)	0.9630 (0.2215)		1.2771* (0.1819)	1.0768 (0.1976)	0.9502 (0.2328)
AI benefits exceed risks		1.2767 * (0.1844)	1.4620 ** (0.2474)	1.4361 (0.3521)		1.2806* (0.1861)	1.4754** (0.2543)	1.4633 (0.3818)
Awareness of AI products		0.8084 * (0.0889)	0.6785 ** (0.0920)	0.5535 ** (0.1002)		0.8044** (0.0888)	0.6688** (0.0934)	0.5239** (0.1182)
AI will change future work		1.2132 * (0.1387)	1.4035 ** (0.2098)	1.8236 ** (0.3880)		1.2154* (0.1393)	1.4218** (0.2151)	1.9036** (0.4344)
High cost of equipment		1.4059 ** (0.2156)	1.0690 (0.1834)	1.2508 (0.3108)		1.4051** (0.2167)	1.0685 (0.1858)	1.2564 (0.3273)
Cost of maintenance		0.8254 (0.1204)	1.0155 (0.1634)	1.0516 (0.2629)		0.8286 (0.1219)	1.0234 (0.1677)	1.0779 (0.2865)
Decisions take time		0.8056 (0.1141)	0.7008 ** (0.1220)	0.8361 (0.1789)		0.8090 (0.1148)	0.7030** (0.1235)	0.8210 (0.1900)
Usability concerns		1.3447 ** (0.1422)	1.2296 * (0.1537)	1.2119 (0.2037)		1.3469*** (0.1425)	1.2295* (0.1549)	1.2308 (0.2285)
Loss of farmer knowledge		1.0989 (0.1169)	1.1362 (0.1496)	0.9654 (0.1863)		1.0961 (0.1168)	1.1336 (0.1507)	0.9497 (0.1941)
Data use uncertainty		1.3976 ** (0.1444)	1.3413 ** (0.1639)	1.5297 ** (0.2494)		1.3977*** (0.1446)	1.3467** (0.1664)	1.5604** (0.2623)
Trust in recommendations		0.6234 ** (0.0846)	0.6550 ** (0.1080)	0.6086 ** (0.1211)		0.6206*** (0.0845)	0.6504** (0.1084)	0.5952** (0.1271)
Validation concerns		0.8826 (0.1488)	0.8178 (0.1784)	0.8471 (0.2209)		0.8805 (0.1482)	0.8151 (0.1796)	0.8325 (0.2353)
Farm size concerns		1.0582 (0.1440)	1.3818 * (0.2327)	1.0380 (0.2570)		1.0575 (0.1445)	1.3835* (0.2356)	1.0322 (0.2680)
Lack of transparency		0.9809 (0.0981)	0.8284 (0.0978)	0.8171 (0.1231)		0.9799 (0.0978)	0.8215* (0.0991)	0.8030 (0.1274)
Corporate misuse of power		0.8574 (0.1636)	0.8733 (0.1884)	1.1083 (0.3117)		0.8577 (0.1633)	0.8757 (0.1907)	1.1170 (0.3214)
Data privacy concerns		0.5944 ** (0.1302)	0.7743 (0.2148)	0.9689 (0.3318)		0.5885** (0.1295)	0.7724 (0.2165)	0.9756 (0.3447)
Corporate trust concern		1.2528 (0.1779)	1.1075 (0.1882)	1.0824 (0.2084)		1.2569 (0.1805)	1.1095 (0.1902)	1.0709 (0.2182)
Regulatory misuse concern		1.0800 (0.1220)	1.1638 (0.1740)	0.8025 (0.1710)		1.0883 (0.1260)	1.1666 (0.1775)	0.7962 (0.1760)
Like engagement with DSS		0.7432 (0.1641)	0.8510 (0.2335)	0.7542 ** (0.2739)		0.7541 (0.1703)	0.8604 (0.2411)	0.7553 (0.2919)
Like testing DSS functions		1.5601 * (0.3706)	1.7624 ** (0.4929)	2.3162** (0.9828)		1.5363* (0.3716)	1.7671** (0.4997)	2.4036** (1.0238)
Use DSS out of obligation		0.8848 (0.1647)	0.6781 * (0.1479)	0.9613 (0.2969)		0.8861 (0.1657)	0.6763* (0.1497)	0.9445 (0.3137)
Like intensive use of new DSS		0.9450 (0.1752)	1.1123 (0.2863)	0.6678 (0.2123)		0.9419 (0.1752)	1.1024 (0.2875)	0.6378 (0.2235)
Enjoy learning new DSS		1.1688 (0.2335)	1.3279 (0.3337)	0.9617 (0.2777)		1.1705 (0.2334)	1.3306 (0.3389)	0.9485 (0.2936)
Only cares if DSS works		1.2259 (0.2366)	1.4899 * (0.3505)	1.1321 (0.3490)		1.2299 (0.2375)	1.4939* (0.3603)	1.1347 (0.3673)
Seeks detailed understanding of DSS		0.8250 (0.1579)	1.0152 (0.2581)	0.8597 (0.2877)		0.8276 (0.1584)	1.0202 (0.2625)	0.8610 (0.3063)
Wants basic functional knowledge of DSS		1.3598 * (0.2533)	1.3669 (0.3109)	1.3483 (0.3920)		1.3573* (0.2529)	1.3736 (0.3176)	1.3767 (0.4322)
Aims to fully use DSS		1.0144 (0.2125)	0.9011 (0.2265)	1.8684** (0.5914)		1.0149 (0.2135)	0.9105 (0.2279)	1.9692* (0.6998)
Farm size by acres		1.0000 ** (0.0000)	0.9999 (0.0000)	0.9999 (0.0000)		1.0000** (0.0000)	0.9999 (0.0000)	0.9999 (0.0000)
Experience by year		0.9885 (0.0140)	0.9797 (0.0177)	0.9791 (0.0260)		0.9883 (0.0141)	0.9791 (0.0180)	0.9771 (0.0271)
Age group		0.9494 (0.1157)	1.0743 (0.1578)	1.1098 (0.2563)		0.9489 (0.1162)	1.0780 (0.1614)	1.1276 (0.2739)
Gender (Male = 0, Female = 1)		1.2111 (0.3972)	1.3539 (0.5640)	0.9624 (0.5391)		1.2223 (0.4046)	1.3555 (0.5730)	0.9675 (0.5810)
Advanced Degree		1.0433 (0.2373)	0.8199 (0.2340)	0.5078 (0.2173)		1.0392 (0.2362)	0.8010 (0.2331)	0.4787* (0.2113)
Race		1.2704 (0.3547)	1.4231 (0.5311)	0.7459 (0.3053)		1.2714 (0.3549)	1.4133 (0.5350)	0.7171 (0.3258)
Self Employed		1.7571 * (0.5481)	1.2708 (0.5319)	1.3690 (0.7258)		1.7648* (0.5508)	1.2738 (0.5400)	1.3854 (0.7722)
Employed by Ag establishment		1.5955 (0.6997)	1.5245 (0.7957)	2.0362 (1.4760)		1.6044 (0.7069)	1.5416 (0.8161)	2.1851 (1.7403)
Employed by Agribusiness		1.3595 (0.3681)	1.7850 (0.6530)	2.1135 (0.8268)		1.3543 (0.3682)	1.6830 (0.6768)	2.2498 (1.2609)
Constant		0.2428 (0.3413)	0.0227 ** (0.0416)	0.0123 (0.0352)		0.2386 (0.3365)	0.0201** (0.0378)	0.0180 (0.0306)
Model specific parameters								
Wald chi^2^ (DF)	189.84*** (4)	449.77*** (18)			162.96*** (4)	325.96*** (18)		
Log (pseudo)likelihood	−4665.327	−2523.3497			−4664.927	−2522.9		
No. of observations	15,292	9,380			15,292	9,380		
No. of cases	3,823	2,345			3,823	2,345		
No. of participants	771	472			771	472		

*** shows significance at 99% level (*p <* 0.001), ** at 95% level (0.001 ≤ *p <* 0.05), and * at 90% level (0.05 ≤ *p <* 0.1).

## 3 Results

### 3.1 Choice models

Table 3 presents estimated coefficients and model fitness statistics from all four models. All four models, M1 to M4 shown in Table 3, provide comparable results for estimated coefficients; however, Model M4 provides more robust estimates due to both the lowest Akaike Information Criterion (AIC) score as well as the model’s ability to account for randomness in the underlying utility functions of the survey respondents. Similar to Table 3, the Table 4 presents predictive margins that are derived for predicting the mean probability of choice of AI-DSS from each of the four, M1 to M4, models. From both Models M4 and M2, while holding all controlling variables constant at their mean values, we find that 25.54% (*p <* 0.001) of CCAs opt out, 45.36% (*p <* 0.001) prefer AI-DSS 1, 19.23% (*p <* 0.001) prefer AI-DSS 2 and 9.85% (*p <* 0.001) prefer AI-DSS 3 (Table 4).

**Table 4:** Predictive margins of AI-DSS choice set derived from all four estimated models.

AI-DSS choice set	M1: standard McFadden logit reduced model margin (s.e.)	M2: standard McFadden logit full model margin (s.e.)	M3: random utility mixed logit reduced model margin (s.e.)	M4: random utility mixed logit full model margin (s.e.)
Opt out	0.2602*** (0.0130)	0.2554*** (0.0138)	0.2602*** (0.0130)	0.2554*** (0.0138)
AI-DSS1	0.4559*** (0.0135)	0.4537*** (0.0155)	0.4559*** (0.0135)	0.4536*** (0.0155)
AI-DSS2	0.1893*** (0.0106)	0.1923*** (0.0126)	0.1894*** (0.0106)	0.1923*** (0.0126)
AI-DSS3	0.0944*** (0.0087)	0.0985*** (0.0109)	0.0944*** (0.0087)	0.0985*** (0.0109)

Standard errors are reported in ()s. *** shows significance at 99% level (*p <* 0.001), ** at 95% level (0.001 ≤ *p <* 0.05), and * at 90% level (0.05 ≤ *p <* 0.1).

The odds ratios for coefficients and their respective standard errors reported in Table 3 provide interpretable information about the predicted change in both attributes of AI-DSS and respondent-specific attributes. The negative coefficient on cost (odds ratio <1) is consistent with the well-established finding about marginal diminishing returns in DCE literature (Holmes et al., 2017). We find that as average cost of AI-DSS increases by 1$/acre/year, the average likelihood of adoption of AI-DSS decreases by 3.86% (*p <* 0.001). Further, we find that for each 1 % increase in the predictive accuracy of AI-DSS, its likelihood of adoption increases by 4.41% (*p <* 0.001). We find a relatively weaker effect size for spatial precision. As the spatial precision of AI-DSS prediction increases by one meter^2^ (note that granular spatial resolution has smaller square meter pixels), its likelihood of adoption decreases by 1.44% (*p <* 0.01).

### 3.2 Hypotheses testing

**Figure 5:**
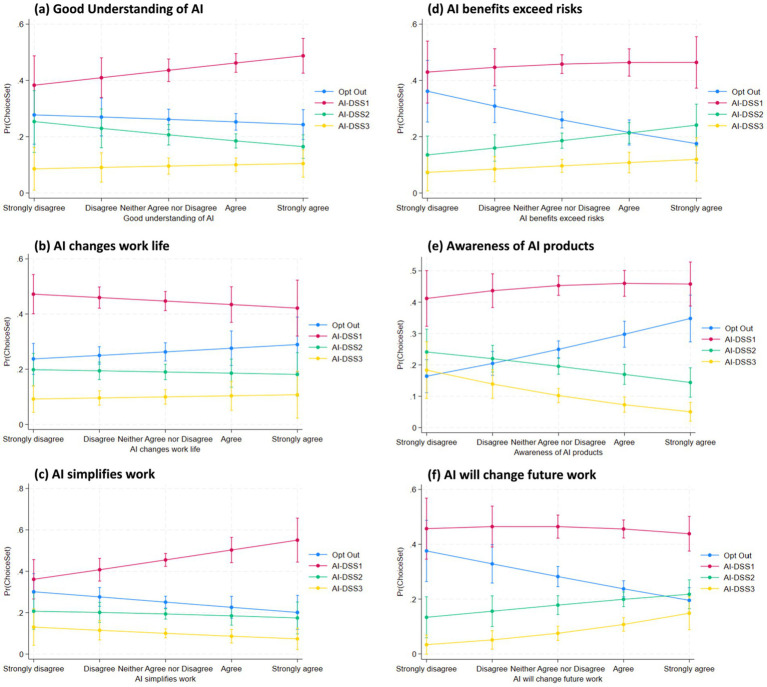
Predictive margins with 95% confidence intervals are derived from random utility mixed logit full model (M4 in Table 3). Predicted probability of adopting AI-DSS choice set (y-axis) vis-à-vis CCA position (strongly disagree to strongly agree on x-axis) are plotted for six statistically significant AI perception items: **(a)** Good understanding of AI; **(b)** AI changes work life; **(c)** AI simplifies work; **(d)** AI benefits exceed risks; **(e)** Awareness of AI products; and **(f)** AI will change future work.

**Figure 6:**
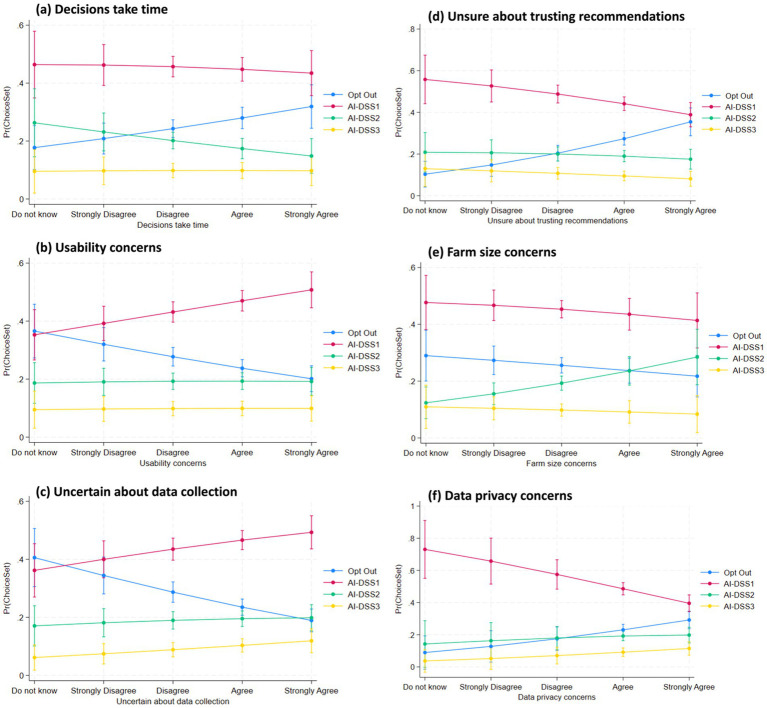
Predictive margins with 95% confidence intervals are derived from random utility mixed logit full model (M4 in Table 3). Predicted probability of adopting AI-DSS choice set (y-axis) vis-à-vis CCA position (do not know to strongly agree on x-axis) are plotted for six statistically significant PA concern items: **(a)** Decisions take time; **(b)** Usability concerns; **(c)** Uncertain about data collection; **(d)** Unsure about trusting recommendations; **(e)** Farm size concerns; and **(f)** Data privacy concerns.

**Figure 7:**
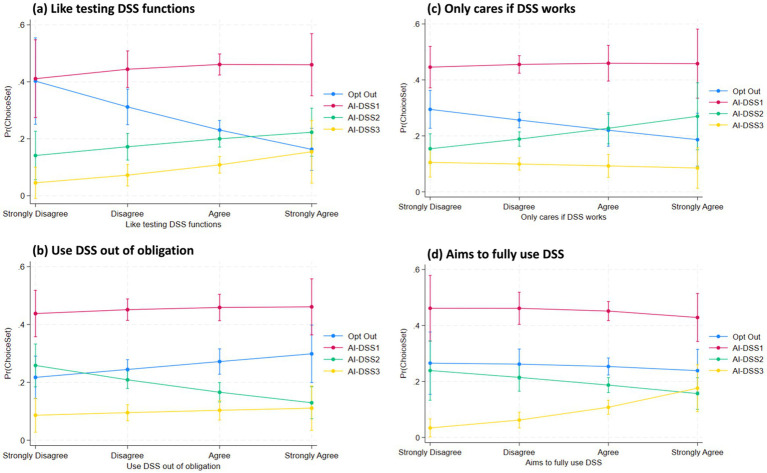
Predictive margins with 95% confidence intervals are derived from random utility mixed logit full model (M4 in Table 3). Predicted probability of adopting AI-DSS choice set (y-axis) vis-à-vis CCA position (strongly disagree to agree on x-axis) are plotted for four statistically significant DSS experience items: **(a)** Like testing DSS functions; **(b)** Use DSS out of obligation; **(c)** Only cares if DSS works; and **(d)** Aims to fully use DSS.

**Figure 8:**
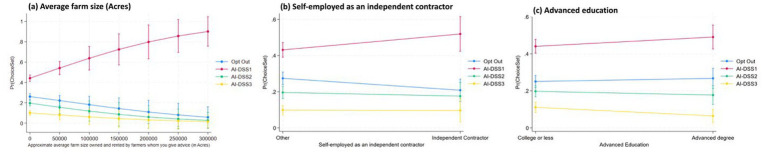
Predictive margins with 95% confidence intervals are derived from random utility mixed logit full model (M4 in Table 3). Predicted probability of adopting AI-DSS choice set (y-axis) vis-à-vis CCA reported average farm size **(a)**, self-employed as independent contractor **(b)** and advance education **(c)** are plotted.

We analyzed crop advisors’ preferences for three AI-based decision support systems (AI-DSS 1, 2, and 3) versus opting out, using a Random Utility Mixed Logit model (Model 4) with odds ratios reported. Key findings are discussed below according to our hypotheses and contrasted with McFadden Logit results (Model 2) where appropriate. The results from the Full Random Utility Mixed Logit Model (Model 4) provide important insights into crop advisors’ preferences for different types of AI decision support systems (AI-DSS), particularly in relation to their beliefs, attitudes, and concerns. The findings are reported as odds ratios (OR) with robust standard errors (SE), and all interpretations focus on significant predictors influencing the likelihood of selecting AI-DSS 1 (low-cost, low-precision), AI-DSS 2 (moderate cost and performance), or AI-DSS 3 (high-end, advanced system), relative to opting out. To further illustrate the effect magnitudes and directions predicted by M4, Figures 5–8 present predictive margins for statistically significant effects of six AI perception survey items (Figure 5), six PA Concern (Figure 6), four DSS experience (Figure 7) and three socio-demographic characteristics of CCAs (Figure 8).

#### 3.2.1 *H1:* crop advisors are generally more likely to choose some form of AI-DSS over opting out entirely, even if it is a lower-performing tool like AI-DSS 1, indicating broad baseline interest

There is partial support for hypothesis H1, which posited that crop advisors would generally prefer adopting some form of AI-DSS over opting out altogether. According to Model 3, the Random Utility Mixed Logit (Reduced Model), the alternative-specific constants (ASCs) for all three AI-DSS options are statistically significant. The ASC represents the average effect on utility of all factors not explicitly included as attributes in the model. The odds of selecting AI-DSS 1 over opting out are 1.235 (SE = 0.0987, *p <* 0.001), while AI-DSS2 and AI-DSS3 have lower odds ratios of 0.3903 (SE = 0.0803, *p <* 0.001) and 0.4148 (SE = 0.0893, *p <* 0.001), respectively. A greater than one odds ratio ASC for AI-DSS1 indicates that this AI-DSS alternative is, on average, preferred compared to the reference alternative or opt-out. A less than one ASC (odds ratio) for AI-DSS 2 and AI-DSS 3 suggests the opposite. Crop advisors are more likely to choose AI-DSS 1 (low-cost, low-precision) than opting-out and are less likely to choose the more intermediate and advanced AI-DSS compared to the reference alternative (opting-out). The McFadden Logit results in Model 1 (Reduced Model) shows the same trend, although odds ratios are somewhat higher, reinforcing the robustness of this finding.

#### 3.2.2 *H2:* crop advisors who believe AI will transform future agricultural work are more likely to prefer higher-performing AI systems, including moderately advanced (AI-DSS 2) and cutting-edge (AI-DSS 3) options

The results (Table 3 and Figure 5) show clear trend in support of H2, which proposed that belief in AI’s transformative impact on agriculture work would increase crop advisors’ preference for more advanced AI-DSS. Respondents who strongly believed AI will reshape future agricultural work (“AI will change future work”) were significantly more likely to select AI-DSS 2 (OR = 1.4218, SE = 0.2151, *p <* 0.05) and AI-DSS 3 (OR = 1.9036, SE = 0.4344, *p <* 0.01). This indicates that transformational beliefs about AI are associated with nearly twice as many odds of choosing the state-of-the-art AI-DSS over opting out.

#### 3.2.3 *H3:* higher costs reduce the likelihood of selecting tools, particularly the most advanced (AI-DSS 3), reflecting economic constraints among potential users

This hypothesis tests the role of cost in shaping AI-DSS preferences. Results (Table 4) show that cost has a consistently negative effect on AI-DSS selection. The odds ratio for the cost variable (main effect) in Model 4 is 0.9614 (SE = 0.0083, *p <* 0.001), meaning that for every unit increase in cost ($/acre/year), the odds of selecting an AI-DSS option decrease by roughly 3.4%. While the direction of this result supports H2, the main effects do not disaggregate this effect across the three AI-DSS. The respondent specific predictor “high cost of equipment” that measures crop advisors concern about the cost of AI-DSS showed that the low-cost option of AI-DSS1 was most sensitive to cost (OR = 1.4051, SE = 0.2167, *p <* 0.05). No significant cost effects were observed for AI-DSS2 or the most advanced AI-DSS 3.

#### 3.2.4 *H4:* crop advisors already familiar with AI products may be more critical or selective, avoiding “in-between” systems like AI-DSS 2, in favor of either simpler (AI-DSS 1) or more sophisticated alternatives (AI-DSS 3)

H4 hypothesized that crop advisors already familiar with AI products would be more selective, particularly avoiding mid-range options like AI-DSS 2. The results (Table 4 and Figure 5) do not support this hypothesis. Instead, awareness of AI significantly decreases the likelihood of selecting AI-DSS 2 (OR = 0.6688, SE = 0.0934, *p <* 0.05) and AI-DSS 3 (OR = 0.5239, SE = 0.1182, *p <* 0.01). The likelihood of selecting the low-cost AI-DSS1 is also negative (OR = 0.8084, SE = 0.0889, 0.1 < *p <* 0.05), but remains higher compared to the in-between and advanced AI-DSS. This suggests that informed users may be more discerning, possibly viewing all AI-DSS with skepticism. In addition, more AI-aware crop advisors may be viewing AI-DSS 2 as insufficiently advanced, or perceiving AI-DSS 3 as overengineered or not cost-effective.

#### 3.2.5 *H5:* lack of trust in AI recommendations significantly reduces preference for all DSS types, with the greatest sensitivity likely in advanced systems (AI-DSS 2 and 3) due to their complexity

Concerns about trust (“Trust in recommendations”) emerged as a strong factor influencing AI-DSS preferences, supporting H5. Crop advisors who expressed a lack of trust in AI recommendations were significantly less likely to choose any AI-DSS option (Table 3 and Figure 6). The odds ratios were 0.6206 (SE = 0.0845, *p <* 0.001) for AI-DSS1, 0.6504 (SE = 0.1084, *p <* 0.05) for AI-DSS2, and 0.5952 (SE = 0.1271, *p <* 0.05) for AI-DSS3. These findings show that trust concerns cut across all system types, with particularly strong aversion toward the more complex systems, highlighting the centrality of trust in decision support system adoption.

#### 3.2.6 *H6:* crop advisors who express usability concerns tend to prefer more basic systems like AI-DSS1, indicating that interface simplicity may outweigh model performance for these users

Regarding usability concerns, H6 proposed that advisors preferring simpler systems would gravitate toward AI-DSS 1. The results (Table 3 and Figure 6) partially support this hypothesis. Respondents reporting usability concerns (“Useability concerns”) were significantly more likely to choose the basic system (AI-DSS 1), with an odds ratio of 1.3469 (SE = 0.1425, *p <* 0.001) and the intermediate system, AI-DSS 2 (OR = 1.2295, SE = 0.1549, <0.1*p <* 0.05). The relationship was not significant for AI-DSS 3, suggesting that simplicity and ease-of-use are particularly important for lower-and intermediate tier AI-DSS.

#### 3.2.7 *H7:* crop advisors who enjoy experimenting with tools are much more likely to prefer advanced systems, especially AI-DSS 3, reflecting alignment with their desire to explore and understand

In line with H7, crop advisors who enjoy experimenting with new tools were significantly more likely to prefer advanced AI-DSS options. Those who reported enthusiasm for testing new DSS features (“Like testing DSS functions”) were 1.53 times more likely to choose AI-DSS 1 (OR = 1.5363, SE = 0.3716, 0.1 < *p <* 0.05), 1.77 times more likely to choose AI-DSS 2 (OR = 1.7671, SE = 0.4997, *p <* 0.05) and 2.40 times more likely to prefer AI-DSS 3 (OR = 2.4036, SE = 1.0238, *p <* 0.05). Additionally, those who stated they want to fully use all DSS functions (“Aims to fully use DSS”) were significantly more likely to prefer AI-DSS 3 (OR = 1.9692, SE = 0.6998, *p <* 0.01). These results (Table 3 and Figure 7) confirm that exploratory and engaged users are drawn to higher-functioning and feature-rich AI systems.

#### 3.2.8 *H8:* data privacy concerns reduce preference for AI-DSS 1, potentially due to perceived lack of transparency or security in low-cost systems

Lastly, H8 examined the role of privacy concerns in determining preferences for AI-DSS. The results (Tabe 3 and Figure 6) indicate that such concerns significantly decrease the odds of selecting the most basic tool, AI-DSS 1 (OR = 0.5885, SE = 0.1295, *p <* 0.05). This suggests that respondents may associate low-cost tools with less data security or transparency measures. However, privacy concerns were not significantly associated with the selection of AI-DSS 2 or AI-DSS 3.

In summary, all eight hypotheses received at least partial support, with particularly robust findings around transformational beliefs, trust, usability, and experimental mindsets. These results offer insight into how both individual-level attitudes and system-level attributes shape the emerging adoption landscape for AI-DSS in agriculture. Among the socio-demographic variables tested in M4, we found that AI-DSS1 was strongly preferred by CCAs managing larger farm size area, self-employed as independent contractors and advanced degree holders (Figure 8).

## 4 Discussion and conclusions

The results of our study offer several critical insights into the factors shaping crop advisors’ preferences for AI-DSS, emphasizing the multifaceted interplay between cost, accuracy, trust, usability, data privacy, and user experience. Drawing from the statistically significant results in Models 4 and 2, and situating them within the broader academic and policy discourse on AI adoption in agriculture, we reflect on the implications for future AI-DSS development, governance, and AI knowledge dissemination.

First, the finding that crop advisors who believe AI will transform future agricultural work are significantly more likely to prefer intermediate and advanced systems (AI-DSS 2 and 3) highlights the influence of techno-optimism in shaping adoption preferences. These individuals may perceive AI not merely as a decision aid but as a transformative force in agri-food systems. This aligns with literature showing that belief in the transformative potential of AI encourages openness to its adoption, particularly for more precise and complex tools that promise improved outcomes for sustainability and productivity (Adereti et al., 2024; Gardezi et al., 2024b).

Second, and consistent with prior research, cost continues to be a significant barrier to AI adoption (Brugler et al., 2024; Khanna, 2021). Our finding that higher AI-DSS cost significantly reduces the odds of selecting AI-DSS 2 and 3 supports earlier studies documenting the financial constraints faced by users, especially those on smaller farms with limited economies of scale (Isgin et al., 2008; Pignatti et al., 2015). As others have noted, these constraints are compounded by long payback periods and uncertainty around economic returns (Lee et al., 2021), making affordability a central concern in AI-DSS design and policy support.

Importantly, even the least advanced system (AI-DSS 1) was more likely to be selected over opting out entirely, reinforcing the baseline interest in AI-DSS among crop advisors. This suggests a broad openness to AI tools despite concerns over costs and capabilities, resonating with previous findings that while users are cautious, they are generally curious and exploratory when it comes to digital innovations (Zhang and Schwarz, 2012; Omrani et al., 2022).

An intriguing pattern emerged with respect to prior experience: crop advisors familiar with AI were less likely to prefer the moderately or fully advanced AI-DSS 2 and AI-DSS 3. This selective preference may reflect a confidence trade-off, where greater technical familiarity leads to sharper evaluations and a more discerning approach. These users may either be drawn to the simplicity of AI-DSS 1 rather than the state-of-the-art performance of AI-DSS 2 & 3, because of viewing the more advanced systems as insufficiently advanced, or too futuristic, or perceiving them as overengineered or not cost-effective. More research is needed to fully unpack the factors that may be driving this confidence trade-off in decision making.

Trust, long recognized as a linchpin in AI adoption (Afroogh et al., 2024; Floridi, 2019; Gardezi and Stock, 2021), was significantly and negatively associated with preferences for all three AI-DSS systems. This effect was especially strong for AI-DSS 2 and 3, likely due to the increased complexity and “black box” nature of advanced systems (Bayer et al., 2022). These findings echo prior work emphasizing that as AI systems become more opaque, they require stronger design efforts to ensure explainability and user confidence (Kim et al., 2021; Winfield and Jirotka, 2018). Providing transparent, interpretable outputs tailored to users’ cognitive levels and technical training is crucial for fostering acceptance (Bhatti et al., 2021).

Usability concerns similarly discouraged preferences for advanced AI-DSS 2 and 3, while being positively associated with AI-DSS 1. This finding reinforces the insight that users are not simply selecting tools based on accuracy but are weighing the trade-offs between complexity and practical usability. Particularly among advisors who lack time or training to engage deeply with high-tech systems, intuitive and accessible interfaces can determine adoption trajectories (Gardezi et al., 2024b; Hengstler et al., 2016).

Advisors who enjoy experimentation were significantly more likely to prefer AI-DSS 3, reflecting an alignment between exploratory disposition and preference for high-performing, cutting-edge tools. This supports findings from user behavior research that suggest innovativeness and curiosity play a role in early adoption, especially when users believe new tools will yield actionable insights (Jain et al., 2018).

The association between data privacy concerns and rejection of AI-DSS 1 suggests a potential contradiction in user expectations. One might assume that lower-cost systems are more appealing to privacy-conscious users, but our results hint that concerns over data ownership and security may be more pronounced when systems are perceived as less robust or transparent. This paradox has been documented by previous research (Idowu et al., 2023; Zhang et al., 2021). This result also speaks directly to the broader idea of “privacy paradox” (Gerber et al., 2018; Solove, 2021), where users report valuing privacy but often trade it off when confronted with systems promising greater utility or ease of use. Responsible data governance, particularly regarding transparency about who owns, uses, and benefits from agricultural data, remains a critical challenge in AI-DSS adoption (Floridi, 2019; Bechtet, 2023; Gardezi et al., 2022).

This study provides empirical evidence that crop advisors’ preferences for AI-DSS are shaped by a mix of techno-optimism, cost sensitivity, prior experience, personal innovativeness, trust, usability and data privacy attitudes. While there is baseline support for AI tools even at low performance levels, advisors are highly sensitive to cost and trust considerations when selecting more advanced systems. These findings highlight the need for differentiated design, communication, and policy strategies to promote AI-DSS adoption. We discovered that 74.4% of the crop advisors sampled participants have statistically significant willingness-to-pay and adopt AI-DSS. For developers, this means focusing not just on improving accuracy and precision, but also ensuring usability, transparency, and ethical data governance. For policymakers and funders, this means recognizing the financial and cognitive barriers that limit uptake, particularly among smaller farms or less tech-savvy users. As AI-DSS continues to evolve, its adoption will depend as much on how these systems are embedded in social and institutional contexts as on their technical sophistication.

Improvements in accuracy and precision of AI-DSS, complemented with a privacy enhancing data ownership design of AI-DSS will have the largest potential for scaling up AI-DSS to improve agricultural yields while lowering their adverse environmental impacts. With rapidly evolving AI technologies, future research can harness DCEs to both test the effects of additional AI-DSS attributes, such as usability, training requirements and technical support, as well as evaluate the effects of shifting AI perceptions, concerns and experiences on the design of trustworthy AI-DSS. The proposed DCE’s may also be expanded beyond CCAs to farm owners, agribusiness managers and policy makers. Further, as operational AI-DSS are deployed and scaled up in farm management settings, DCE’s can be designed to evaluate and co-configure the features of AI-DSS in operational settings. Future work should also explore how these findings play out across different agricultural contexts and examine the impact of policy interventions, such as subsidies or training programs, on increasing adoption equity. It is only by addressing the full range of social, economic, and ethical dimensions that AI-DSS can realize their transformative potential in agriculture. Our team is launching similar choice experiments for farmers and urban gardeners in the agricultural systems arena. Further, similar choice experiments are being designed to test AI-DSS configurations in water resources and climate change mitigation and adaptation policy designs.

## Data Availability

The datasets presented in this study can be found in online repositories. The names of the repository/repositories and accession number(s) can be found at: https://doi.org/10.7910/DVN/CODYMJ.
