# Supplementary material for: Harnessing discrete choice experiments to elicit preferred configurations of trustworthy AI augmented decision support systems for certified crop advisors
Source: Front Artif Intell. 2026 May 13;9:1747663. doi: 10.3389/frai.2026.1747663 (PMC13212300; doi:10.3389/frai.2026.1747663)
Supplement: Supplementary file 1 [file Data_Sheet_1.DOCX]

**Supplementary Information**

**Harnessing Discrete Choice Experiments to Elicit Preferred Configurations of Trustworthy AI augmented Decision Support Systems (AI-DSS) for Crop Certified Advisors**

**1. Data**

Table S1: Descriptive Statistics

Variable | Obs Mean Std. dev. Min Max

-------------+---------------------------------------------------------

ID_Temp | 15,292 101912 1103.641 100001 103823

ChoiceSet | 15,292 2.5 1.118071 1 4

AIDSS | 15,292 .25 .4330269 0 1

ResponseID | 15,292 1.26e+08 481604.1 1.26e+08 1.28e+08

EXPERIMENT | 15,292 2.997646 1.413795 1 5

-------------+---------------------------------------------------------

ChoiceExpe~t | 15,292 2.159037 1.251491 1 4

ResponseSt~s | 0

IPAddress | 0

Timestampm~y | 15,292 2.03e+12 4.44e+08 2.02e+12 2.03e+12

Duplicate | 15,292 0 0 0 0

-------------+---------------------------------------------------------

TimeTakent~s | 15,292 680.6613 693.4913 45 6140

SeqNumber | 15,292 1 0 1 1

ExternalRe~e | 0

CountryCode | 0

Region | 0

-------------+---------------------------------------------------------

ChoiceExpe~1 | 15,232 1.947216 1.227183 1 4

ChoiceExpe~2 | 15,232 2.317752 1.189488 1 4

ChoiceExpe~3 | 15,244 1.887431 1.205153 1 4

ChoiceExpe~4 | 15,200 2.302105 1.28161 1 4

ChoiceExpe~5 | 15,176 2.340801 1.274083 1 4

-------------+---------------------------------------------------------

Specializa~o | 12,316 1 0 1 1

_v1 | 10,664 1 0 1 1

Specializa~n | 8,220 1 0 1 1

Specializ~es | 5,936 1 0 1 1

Specializ~ns | 8,676 1 0 1 1

-------------+---------------------------------------------------------

Specializa~p | 6,312 1 0 1 1

Specializa~r | 0

AI_Percept~1 | 14,272 3.655269 .9351446 1 5

AI_Percept~2 | 13,872 2.543829 1.040502 1 5

AI_Percept~3 | 13,612 2.950926 .9226661 1 5

-------------+---------------------------------------------------------

AI_Percept~4 | 13,992 3.211549 .8930282 1 5

AI_Percept~5 | 14,032 3.156499 1.004172 1 5

AI_Percept~6 | 14,052 3.669798 .9699735 1 5

PA_Concern1 | 14,252 2.680606 1.0423 0 4

PA_Concern2 | 14,272 2.538677 1.130124 0 4

-------------+---------------------------------------------------------

PA_Concern3 | 14,232 2.313097 .9147807 0 4

PA_Concern4 | 14,252 2.504631 1.097413 0 4

PA_Concern5 | 14,272 1.939182 .921531 0 4

PA_Concern6 | 14,212 2.5736 1.094893 0 4

PA_Concern7 | 14,212 2.650155 .9558758 0 4

-------------+---------------------------------------------------------

PA_Concern8 | 14,272 3.368834 .7256407 0 4

PA_Concern9 | 14,252 1.959585 .8387132 0 4

PA_Concern10 | 14,252 2.854617 1.164667 0 4

PA_Concern11 | 14,272 3.266256 .8626601 0 4

PA_Concern12 | 14,252 3.248106 .864178 0 4

-------------+---------------------------------------------------------

PA_Concern13 | 14,252 3.191973 1.020826 0 4

PA_Concern14 | 14,212 3.180692 1.008304 0 4

DSS_Experi~1 | 13,556 2.61198 .6275969 1 4

DSS_Experi~2 | 13,516 2.691921 .6340422 1 4

DSS_Experi~3 | 13,536 2.386229 .6274589 1 4

-------------+---------------------------------------------------------

DSS_Experi~4 | 13,496 2.457321 .6563406 1 4

DSS_Experi~5 | 13,536 2.53487 .6845998 1 4

DSS_Experi~6 | 13,516 2.073394 .6807535 1 4

DSS_Experi~7 | 13,536 2.872636 .6418725 1 4

DSS_Experi~8 | 13,516 2.473217 .6614653 1 4

-------------+---------------------------------------------------------

DSS_Experi~9 | 13,516 2.743119 .6346809 1 4

FarmSize_A~s | 10,860 5737.134 21253.22 0 300000

Experience~s | 12,796 21.97999 79.19674 0 2000

Education | 13,436 4.168205 .6749192 1 5

Gender | 13,416 1.215564 .571962 1 4

-------------+---------------------------------------------------------

Race | 13,376 10.49103 1.647494 1 11

Income | 13,336 4.80114 1.447303 1 7

Employment~s | 13,416 2.54025 1.018804 1 4

ZipCode | 0

AgeGroup | 13,376 3.850478 1.469566 1 6

-------------+---------------------------------------------------------

ResponseSt~m | 15,292 .8681664 .3383209 0 1

AIDSS0 | 15,292 .2602668 .4387945 0 1

AIDSS1 | 15,292 .4559247 .4980699 0 1

AIDSS2 | 15,292 .1893801 .391823 0 1

AIDSS3 | 15,292 .0944285 .2924334 0 1

-------------+---------------------------------------------------------

cost | 15,292 23.79976 29.99596 0 80

data | 15,292 1.950366 .8646302 1 3

Female | 13,036 .1374655 .3443513 0 1

AdvancedDe~e | 13,436 .290265 .4539015 0 1

White | 13,376 .860945 .3460169 0 1

-------------+---------------------------------------------------------

QCL_1 | 14,152 1.586772 .4924304 1 2

AI_Percept~C | 14,152 .4132278 .4924304 0 1

QCL_2 | 13,952 1.577408 .4939893 1 2

PAConcern | 13,952 .4225917 .4939893 0 1

QCL_3 | 13,296 1.572202 .494778 1 2

-------------+---------------------------------------------------------

DSSValue | 13,296 .5722022 .494778 0 1

Country_USA | 15,292 .8059116 .3955102 0 1

Data_Compa~y | 15,292 .25 .4330269 0 1

Data_Compa~u | 15,292 .3501831 .4770427 0 1

SelfEmployed | 13,416 .2510435 .4336297 0 1

-------------+---------------------------------------------------------

EmployedAg~t | 13,416 .1013715 .3018313 0 1

EmployedAg~s | 13,416 .503876 .5000036 0 1

accuracy | 15,292 80.40178 10.41452 70 99

precision | 15,292 48.14942 43.26446 .25 100

_v2 | 15,292 1912 1103.641 1 3823

-------------+---------------------------------------------------------

_v3 | 15,292 1541.093 888.9544 1 3084

_v4 | 3,823 2.11797 .9020291 1 4

_caseid | 15,292 1912 1103.641 1 3823

_panelaltid | 15,292 1541.093 888.9544 1 3084

_chosen_al~e | 3,823 2.11797 .9020291 1 4

**2. Methods**

Table S2: Tabulation of choice-set possibilities by EXPERIMENT

| Experiment No (Randomized)

Choice set | 1 2 3 4 5 | Total

-----------+-------------------------------------------------------+----------

1 2 3 4 | 766 765 766 764 762 | 3,823

-----------+-------------------------------------------------------+----------

Total | 766 765 766 764 762 | 3,823

Note: Total is number of cases.

Table S3: Statistics by chosen alternatives (AIDSS = 1)

Summary statistics: Mean

Group variable: _chosen_alternative (AIDSS = 1)

_chosen_alternative | cost accuracy precis~n data

--------------------+----------------------------------------

Opt Out | 0 70 100 1

AI-DSS1 | 1.531842 72.57602 61.87608 2.016638

AI-DSS2 | 20.55249 85 21.57459 2

AI-DSS3 | 70.52632 94.03878 .8691136 3

--------------------+----------------------------------------

Total | 11.25033 76.28512 58.40538 1.841747

-------------------------------------------------------------

.

. cmtab, choice(AIDSS)

Table S4: Tabulation of chosen alternatives (AIDSS = 1)

Choice Set | Freq. Percent Cum.

------------+-----------------------------------

Opt Out | 995 26.03 26.03

AI-DSS1 | 1,743 45.59 71.62

AI-DSS2 | 724 18.94 90.56

AI-DSS3 | 361 9.44 100.00

------------+-----------------------------------

Total | 3,823 100.00

**3. Extended Results**

Table S5. McFadden reduced model (M1)

. cmclogit AIDSS cost accuracy precision data, basealternative(1) or

note: data were cmset as panel data, and the default vcetype for panel data is vce(cluster ResponseID); see cmclogit.

Iteration 0: Log pseudolikelihood = -4737.8006

Iteration 1: Log pseudolikelihood = -4665.8223

Iteration 2: Log pseudolikelihood = -4665.3271

Iteration 3: Log pseudolikelihood = -4665.327

Conditional logit choice model Number of obs = 15,292

Case ID variable: _caseid Number of cases = 3823

Alternatives variable: ChoiceSet Alts per case: min = 4

avg = 4.0

max = 4

Wald chi2(4) = 189.84

Log pseudolikelihood = -4665.327 Prob > chi2 = 0.0000

(Std. err. adjusted for 771 clusters in ResponseID)

------------------------------------------------------------------------------

| Robust

AIDSS | Odds ratio std. err. z P>|z| [95% conf. interval]

-------------+----------------------------------------------------------------

ChoiceSet |

cost | .9736524 .0030508 -8.52 0.000 .9676912 .9796504

accuracy | 1.031649 .0072305 4.45 0.000 1.017575 1.045919

precision | .9922684 .0043435 -1.77 0.076 .9837917 1.000818

data | 1.109234 .1917611 0.60 0.549 .790442 1.556598

-------------+----------------------------------------------------------------

Opt_Out | (base alternative)

-------------+----------------------------------------------------------------

AI_DSS1 |

_cons | 1.238113 .0990296 2.67 0.008 1.058468 1.448248

-------------+----------------------------------------------------------------

AI_DSS2 |

_cons | .3992724 .081074 -4.52 0.000 .2681816 .594442

-------------+----------------------------------------------------------------

AI_DSS3 |

_cons | .4303737 .0905742 -4.01 0.000 .2849091 .6501075

------------------------------------------------------------------------------

Note: _cons estimates baseline relative risk for each outcome.

.

. margins

Predictive margins Number of obs = 15,292

Model VCE: Robust

Expression: Pr(ChoiceSet|1 selected), predict()

------------------------------------------------------------------------------

| Delta-method

| Margin std. err. z P>|z| [95% conf. interval]

-------------+----------------------------------------------------------------

_outcome |

Opt Out | .2602668 .0130007 20.02 0.000 .2347859 .2857477

AI-DSS1 | .4559247 .013518 33.73 0.000 .4294299 .4824195

AI-DSS2 | .1893801 .0106218 17.83 0.000 .1685618 .2101984

AI-DSS3 | .0944285 .008728 10.82 0.000 .0773219 .111535

------------------------------------------------------------------------------

Table S6. McFadden full model (M2)

. cmclogit AIDSS cost accuracy precision data, casevars(AI_Perception1 AI_Perception2 AI_Perception3 AI_Perception4 AI_Perception5 AI_Perception6 PA_Concern1 PA_Concern2 PA_Concern3 PA_Concern4 PA_Con

> cern5 PA_Concern6 PA_Concern7 PA_Concern8 PA_Concern9 PA_Concern10 PA_Concern11 PA_Concern12 PA_Concern13 PA_Concern14 DSS_Experience1 DSS_Experience2 DSS_Experience3 DSS_Experience4 DSS_Experience5

> DSS_Experience6 DSS_Experience7 DSS_Experience8 DSS_Experience9 FarmSize_Acres Experience_Years AgeGroup Female AdvancedDegree White SelfEmployed EmployedAgEstablishment EmployedAgriBusiness) basea

> lternative(1) or

note: data were cmset as panel data, and the default vcetype for panel data is vce(cluster ResponseID); see cmclogit.

Iteration 0: Log pseudolikelihood = -2658.3178

Iteration 1: Log pseudolikelihood = -2528.6937

Iteration 2: Log pseudolikelihood = -2523.3769

Iteration 3: Log pseudolikelihood = -2523.3497

Iteration 4: Log pseudolikelihood = -2523.3497

Conditional logit choice model Number of obs = 9,380

Case ID variable: _caseid Number of cases = 2345

Alternatives variable: ChoiceSet Alts per case: min = 4

avg = 4.0

max = 4

Wald chi2(118) = 449.77

Log pseudolikelihood = -2523.3497 Prob > chi2 = 0.0000

(Std. err. adjusted for 472 clusters in ResponseID)

-----------------------------------------------------------------------------------------

| Robust

AIDSS | Odds ratio std. err. z P>|z| [95% conf. interval]

------------------------+----------------------------------------------------------------

ChoiceSet |

cost | .9660304 .0041936 -7.96 0.000 .9578461 .9742847

accuracy | 1.045583 .010978 4.25 0.000 1.024287 1.067322

precision | .9887907 .0057675 -1.93 0.053 .9775509 1.00016

data | 1.028274 .2406217 0.12 0.905 .6500149 1.626652

------------------------+----------------------------------------------------------------

Opt_Out | (base alternative)

------------------------+----------------------------------------------------------------

AI_DSS1 |

AI_Perception1 | 1.114211 .1361054 0.89 0.376 .8769799 1.415615

AI_Perception2 | .9066814 .1146165 -0.77 0.438 .7077038 1.161603

AI_Perception3 | 1.274638 .1809657 1.71 0.087 .9650244 1.683587

AI_Perception4 | 1.276756 .1844141 1.69 0.091 .9619682 1.694553

AI_Perception5 | .8084794 .0889774 -1.93 0.053 .6516131 1.003109

AI_Perception6 | 1.213276 .1387145 1.69 0.091 .9697088 1.518022

PA_Concern1 | 1.405912 .2156788 2.22 0.026 1.040822 1.899065

PA_Concern2 | .8254349 .1204123 -1.32 0.188 .6201714 1.098636

PA_Concern3 | .805635 .1141117 -1.53 0.127 .6103409 1.063418

PA_Concern4 | 1.344726 .1422385 2.80 0.005 1.092944 1.654511

PA_Concern5 | 1.098924 .1169624 0.89 0.375 .8920132 1.35383

PA_Concern6 | 1.39761 .1444748 3.24 0.001 1.141288 1.711501

PA_Concern7 | .6234529 .0846779 -3.48 0.001 .4777413 .8136068

PA_Concern8 | .8826177 .1488827 -0.74 0.459 .6341461 1.228446

PA_Concern9 | 1.058218 .1440404 0.42 0.678 .8104252 1.381775

PA_Concern10 | .980963 .0981262 -0.19 0.848 .8063183 1.193435

PA_Concern11 | .857491 .1636504 -0.81 0.420 .5899015 1.246464

PA_Concern12 | .5944703 .1302783 -2.37 0.018 .3868914 .9134215

PA_Concern13 | 1.252887 .1797197 1.57 0.116 .9458268 1.659632

PA_Concern14 | 1.080076 .1220925 0.68 0.496 .865434 1.347953

DSS_Experience1 | .7432389 .1641932 -1.34 0.179 .4820417 1.145967

DSS_Experience2 | 1.560194 .3706931 1.87 0.061 .97935 2.485531

DSS_Experience3 | .884789 .1647084 -0.66 0.511 .6143046 1.27437

DSS_Experience4 | .9450535 .1752155 -0.30 0.761 .6571149 1.359163

DSS_Experience5 | 1.168861 .2335608 0.78 0.435 .7900903 1.729216

DSS_Experience6 | 1.225949 .2366677 1.06 0.291 .8397479 1.789763

DSS_Experience7 | .8250894 .1579668 -1.00 0.315 .5669372 1.20079

DSS_Experience8 | 1.359818 .2533668 1.65 0.099 .9438029 1.959206

DSS_Experience9 | 1.014495 .2125807 0.07 0.945 .6728004 1.529726

FarmSize_Acres | 1.000009 3.74e-06 2.35 0.019 1.000001 1.000016

Experience_Years | .9885517 .0140894 -0.81 0.419 .9613192 1.016556

AgeGroup | .9494308 .1157021 -0.43 0.670 .7477077 1.205576

Female | 1.211149 .3972165 0.58 0.559 .6368421 2.303368

AdvancedDegree | 1.043354 .2373265 0.19 0.852 .6680563 1.629484

White | 1.270467 .3547748 0.86 0.391 .7349661 2.196136

SelfEmployed | 1.757102 .5481302 1.81 0.071 .9533741 3.238399

EmployedAgEstablishment | 1.5955 .6997701 1.07 0.287 .6754118 3.768992

EmployedAgriBusiness | 1.353968 .3681253 1.11 0.265 .7946548 2.306952

_cons | .2428963 .3413474 -1.01 0.314 .01546 3.816214

------------------------+----------------------------------------------------------------

AI_DSS2 |

AI_Perception1 | .9261583 .1433608 -0.50 0.620 .6837967 1.254421

AI_Perception2 | .9157472 .1406841 -0.57 0.567 .6776532 1.237496

AI_Perception3 | 1.081269 .1955 0.43 0.666 .7586324 1.541119

AI_Perception4 | 1.462098 .24742 2.24 0.025 1.049383 2.037131

AI_Perception5 | .6785357 .092044 -2.86 0.004 .5201235 .885195

AI_Perception6 | 1.403528 .2098993 2.27 0.023 1.046943 1.881566

PA_Concern1 | 1.069018 .1834434 0.39 0.697 .7636927 1.496411

PA_Concern2 | 1.015574 .163444 0.10 0.923 .7408351 1.392201

PA_Concern3 | .7008318 .1220235 -2.04 0.041 .4982061 .9858676

PA_Concern4 | 1.229679 .1537966 1.65 0.098 .9623469 1.571274

PA_Concern5 | 1.136237 .1496958 0.97 0.332 .8776585 1.470998

PA_Concern6 | 1.34139 .1639714 2.40 0.016 1.055612 1.704535

PA_Concern7 | .655094 .1080866 -2.56 0.010 .4740897 .9052047

PA_Concern8 | .8178981 .178407 -0.92 0.357 .533369 1.254211

PA_Concern9 | 1.381807 .2327319 1.92 0.055 .9933064 1.922258

PA_Concern10 | .8284985 .0978969 -1.59 0.111 .6572222 1.044411

PA_Concern11 | .8733391 .1884947 -0.63 0.530 .5720924 1.333213

PA_Concern12 | .7743285 .2148138 -0.92 0.357 .4495577 1.333721

PA_Concern13 | 1.107575 .1882177 0.60 0.548 .7938208 1.545338

PA_Concern14 | 1.163822 .1740482 1.01 0.310 .8681416 1.560209

DSS_Experience1 | .8510245 .2335502 -0.59 0.557 .496985 1.457273

DSS_Experience2 | 1.762475 .4929567 2.03 0.043 1.018698 3.049302

DSS_Experience3 | .6781037 .1479966 -1.78 0.075 .4421003 1.040091

DSS_Experience4 | 1.11235 .2863128 0.41 0.679 .6716557 1.842197

DSS_Experience5 | 1.327935 .333749 1.13 0.259 .8114188 2.173245

DSS_Experience6 | 1.488977 .3550662 1.67 0.095 .933056 2.376119

DSS_Experience7 | 1.015226 .2581959 0.06 0.953 .616712 1.671258

DSS_Experience8 | 1.366916 .3109042 1.37 0.169 .8752586 2.13475

DSS_Experience9 | .9015122 .2226504 -0.42 0.675 .55558 1.462839

FarmSize_Acres | .9999992 6.23e-06 -0.12 0.903 .999987 1.000011

Experience_Years | .9797684 .0177645 -1.13 0.260 .945562 1.015212

AgeGroup | 1.074313 .1578516 0.49 0.626 .8054922 1.432847

Female | 1.353932 .5640528 0.73 0.467 .5983887 3.063447

AdvancedDegree | .8197932 .2340154 -0.70 0.486 .4685146 1.43445

White | 1.423126 .5311368 0.95 0.344 .684793 2.957518

SelfEmployed | 1.270807 .531918 0.57 0.567 .5594928 2.886454

EmployedAgEstablishment | 1.524517 .7935842 0.81 0.418 .5495926 4.228862

EmployedAgriBusiness | 1.785024 .6530167 1.58 0.113 .8714621 3.656283

_cons | .0227833 .0416625 -2.07 0.039 .0006325 .8206578

------------------------+----------------------------------------------------------------

AI_DSS3 |

AI_Perception1 | 1.102934 .2436205 0.44 0.657 .7153736 1.70046

AI_Perception2 | .96568 .2060115 -0.16 0.870 .6356877 1.466975

AI_Perception3 | .9630787 .2215655 -0.16 0.870 .6135285 1.511781

AI_Perception4 | 1.436164 .3521161 1.48 0.140 .8881947 2.322203

AI_Perception5 | .5535558 .100239 -3.27 0.001 .3881719 .7894029

AI_Perception6 | 1.823617 .3880913 2.82 0.005 1.201672 2.767459

PA_Concern1 | 1.250823 .3108073 0.90 0.368 .7685768 2.035655

PA_Concern2 | 1.051654 .2629755 0.20 0.840 .6442024 1.716815

PA_Concern3 | .836181 .178981 -0.84 0.403 .5496727 1.272027

PA_Concern4 | 1.211903 .2037991 1.14 0.253 .8716174 1.685038

PA_Concern5 | .9654887 .1863568 -0.18 0.856 .6613782 1.409433

PA_Concern6 | 1.529705 .2494025 2.61 0.009 1.111292 2.105655

PA_Concern7 | .6086044 .1211568 -2.49 0.013 .4119872 .8990553

PA_Concern8 | .8471205 .2209234 -0.64 0.525 .5081097 1.412319

PA_Concern9 | 1.038047 .2570723 0.15 0.880 .6388764 1.686619

PA_Concern10 | .8171839 .1231019 -1.34 0.180 .6082658 1.097858

PA_Concern11 | 1.108354 .3117447 0.37 0.715 .6386494 1.92351

PA_Concern12 | .9689573 .3318821 -0.09 0.927 .4951714 1.896067

PA_Concern13 | 1.08244 .2084458 0.41 0.681 .7421431 1.578774

PA_Concern14 | .8025351 .1710394 -1.03 0.302 .5285093 1.21864

DSS_Experience1 | .7542925 .2739137 -0.78 0.437 .3701984 1.536898

DSS_Experience2 | 2.316261 .9828098 1.98 0.048 1.008359 5.32059

DSS_Experience3 | .961399 .2969508 -0.13 0.899 .5247953 1.761235

DSS_Experience4 | .667892 .2123355 -1.27 0.204 .3581733 1.24543

DSS_Experience5 | .9617027 .2771124 -0.14 0.892 .5467252 1.691658

DSS_Experience6 | 1.13212 .3490705 0.40 0.687 .6186408 2.071793

DSS_Experience7 | .859705 .2877772 -0.45 0.652 .4460861 1.656839

DSS_Experience8 | 1.34836 .392029 1.03 0.304 .7626461 2.383902

DSS_Experience9 | 1.868445 .5914609 1.97 0.048 1.004686 3.474804

FarmSize_Acres | .9999993 7.26e-06 -0.10 0.922 .9999851 1.000014

Experience_Years | .9791495 .0260213 -0.79 0.428 .9294542 1.031502

AgeGroup | 1.109824 .2563386 0.45 0.652 .7057487 1.745251

Female | .9624909 .5391221 -0.07 0.946 .3210786 2.88524

AdvancedDegree | .5078537 .2173124 -1.58 0.113 .2195363 1.174819

White | .7459082 .3051406 -0.72 0.474 .3345574 1.66303

SelfEmployed | 1.369033 .7258559 0.59 0.554 .4842953 3.870059

EmployedAgEstablishment | 2.036211 1.476068 0.98 0.327 .4917891 8.43076

EmployedAgriBusiness | 2.111351 1.082865 1.46 0.145 .7726765 5.769298

_cons | .0123202 .0352095 -1.54 0.124 .0000455 3.336154

-----------------------------------------------------------------------------------------

Note: Exponentiated coefficients represent odds ratios for alternative-specific variables (first equation) and relative-risk ratios for case-specific variables.

Note: _cons estimates baseline relative risk for each outcome.

.

. margins

Predictive margins Number of obs = 9,380

Model VCE: Robust

Expression: Pr(ChoiceSet|1 selected), predict()

------------------------------------------------------------------------------

| Delta-method

| Margin std. err. z P>|z| [95% conf. interval]

-------------+----------------------------------------------------------------

_outcome |

Opt Out | .2554371 .013862 18.43 0.000 .2282681 .2826061

AI-DSS1 | .4537313 .0155566 29.17 0.000 .4232409 .4842217

AI-DSS2 | .1923241 .0126753 15.17 0.000 .1674809 .2171672

AI-DSS3 | .0985075 .0109461 9.00 0.000 .0770534 .1199615

------------------------------------------------------------------------------

Table S7. Random utility reduced model (M3)

. cmmixlogit AIDSS accuracy precision data, random(cost) basealternative(1) or

note: data were cmset as panel data, and the default vcetype for panel data is vce(cluster ResponseID); see cmmixlogit.

Fitting fixed parameter model:

Fitting full model:

Iteration 0: Log simulated-pseudolikelihood = -6272.5585 (not concave)

Iteration 1: Log simulated-pseudolikelihood = -4834.6734 (not concave)

Iteration 2: Log simulated-pseudolikelihood = -4690.0266 (not concave)

Iteration 3: Log simulated-pseudolikelihood = -4669.5124 (not concave)

Iteration 4: Log simulated-pseudolikelihood = -4665.8479

Iteration 5: Log simulated-pseudolikelihood = -4664.9295

Iteration 6: Log simulated-pseudolikelihood = -4664.927

Iteration 7: Log simulated-pseudolikelihood = -4664.927

Mixed logit choice model Number of obs = 15,292

Case ID variable: _caseid Number of cases = 3,823

Alternatives variable: ChoiceSet Alts per case: min = 4

avg = 4.0

max = 4

Integration sequence: Hammersley

Integration points: 759 Wald chi2(4) = 162.96

Log simulated-pseudolikelihood = -4664.927 Prob > chi2 = 0.0000

(Std. err. adjusted for 771 clusters in ResponseID)

------------------------------------------------------------------------------

| Robust

AIDSS | Odds ratio std. err. z P>|z| [95% conf. interval]

-------------+----------------------------------------------------------------

ChoiceSet |

accuracy | 1.030174 .0074324 4.12 0.000 1.015709 1.044845

precision | .9896969 .0047555 -2.16 0.031 .9804201 .9990616

data | 1.020852 .1873895 0.11 0.910 .7123847 1.462887

cost | .9700134 .0043556 -6.78 0.000 .961514 .9785878

-------------+----------------------------------------------------------------

/Normal |

sd(cost)| .0123086 .0059029 .0048083 .0315082

-------------+----------------------------------------------------------------

Opt_Out | (base alternative)

-------------+----------------------------------------------------------------

AI_DSS1 |

_cons | 1.235583 .0987769 2.65 0.008 1.056389 1.445174

-------------+----------------------------------------------------------------

AI_DSS2 |

_cons | .3903136 .0803146 -4.57 0.000 .2607729 .5842046

-------------+----------------------------------------------------------------

AI_DSS3 |

_cons | .4148146 .0893015 -4.09 0.000 .2720239 .632559

------------------------------------------------------------------------------

Note: _cons estimates baseline relative risk for each outcome.

.

. margins

Predictive margins Number of obs = 15,292

Model VCE: Robust

Expression: Pr(ChoiceSet), predict()

------------------------------------------------------------------------------

| Delta-method

| Margin std. err. z P>|z| [95% conf. interval]

-------------+----------------------------------------------------------------

_outcome |

Opt Out | .2602019 .0130039 20.01 0.000 .2347147 .2856891

AI-DSS1 | .4559002 .0135148 33.73 0.000 .4294117 .4823886

AI-DSS2 | .189422 .0106198 17.84 0.000 .1686075 .2102365

AI-DSS3 | .094476 .0087427 10.81 0.000 .0773406 .1116113

------------------------------------------------------------------------------

Table S8. Random utility full model (M4)

. cmmixlogit AIDSS accuracy precision data, casevars(AI_Perception1 AI_Perception2 AI_Perception3 AI_Perception4 AI_Perception5 AI_Perception6 PA_Concern1 PA_Concern2 PA_Concern3 PA_Concern4 PA_Concer

> n5 PA_Concern6 PA_Concern7 PA_Concern8 PA_Concern9 PA_Concern10 PA_Concern11 PA_Concern12 PA_Concern13 PA_Concern14 DSS_Experience1 DSS_Experience2 DSS_Experience3 DSS_Experience4 DSS_Experience5 DS

> S_Experience6 DSS_Experience7 DSS_Experience8 DSS_Experience9 FarmSize_Acres Experience_Years AgeGroup Female AdvancedDegree White SelfEmployed EmployedAgEstablishment EmployedAgriBusiness) random(c

> ost) basealternative(1) or

note: data were cmset as panel data, and the default vcetype for panel data is vce(cluster ResponseID); see cmmixlogit.

Fitting fixed parameter model:

Fitting full model:

Iteration 0: Log simulated-pseudolikelihood = -3527.4103 (not concave)

Iteration 1: Log simulated-pseudolikelihood = -2832.2597 (not concave)

Iteration 2: Log simulated-pseudolikelihood = -2542.1933

Iteration 3: Log simulated-pseudolikelihood = -2531.516

Iteration 4: Log simulated-pseudolikelihood = -2522.9592

Iteration 5: Log simulated-pseudolikelihood = -2522.9

Iteration 6: Log simulated-pseudolikelihood = -2522.9

Mixed logit choice model Number of obs = 9,380

Case ID variable: _caseid Number of cases = 2,345

Alternatives variable: ChoiceSet Alts per case: min = 4

avg = 4.0

max = 4

Integration sequence: Hammersley

Integration points: 703 Wald chi2(118) = 325.96

Log simulated-pseudolikelihood = -2522.9 Prob > chi2 = 0.0000

(Std. err. adjusted for 472 clusters in ResponseID)

-----------------------------------------------------------------------------------------

| Robust

AIDSS | Odds ratio std. err. z P>|z| [95% conf. interval]

------------------------+----------------------------------------------------------------

ChoiceSet |

accuracy | 1.044152 .0112989 3.99 0.000 1.02224 1.066534

precision | .9856634 .0073241 -1.94 0.052 .9714125 1.000123

data | .9341094 .252682 -0.25 0.801 .5497216 1.587277

cost | .9614916 .0083836 -4.50 0.000 .9451995 .9780644

------------------------+----------------------------------------------------------------

/Normal |

sd(cost)| .0142063 .0127757 .0024378 .082788

------------------------+----------------------------------------------------------------

Opt_Out | (base alternative)

------------------------+----------------------------------------------------------------

AI_DSS1 |

AI_Perception1 | 1.116662 .1361314 0.91 0.365 .8793306 1.418048

AI_Perception2 | .9063552 .1148597 -0.78 0.438 .7070143 1.1619

AI_Perception3 | 1.277169 .1819579 1.72 0.086 .9660014 1.688569

AI_Perception4 | 1.280682 .1861047 1.70 0.089 .9632682 1.702688

AI_Perception5 | .8044025 .0888927 -1.97 0.049 .6477524 .9989362

AI_Perception6 | 1.215486 .139231 1.70 0.088 .9710621 1.521434

PA_Concern1 | 1.405126 .2167977 2.20 0.027 1.038444 1.901287

PA_Concern2 | .8286189 .1219513 -1.28 0.201 .6209833 1.105681

PA_Concern3 | .8090249 .114837 -1.49 0.135 .6125451 1.068527

PA_Concern4 | 1.346992 .1425633 2.81 0.005 1.09465 1.657504

PA_Concern5 | 1.096197 .1168925 0.86 0.389 .8894491 1.351002

PA_Concern6 | 1.397302 .1446368 3.23 0.001 1.140725 1.711589

PA_Concern7 | .6206277 .0845692 -3.50 0.000 .4751635 .8106237

PA_Concern8 | .880513 .1482108 -0.76 0.450 .6330803 1.224652

PA_Concern9 | 1.05756 .1445443 0.41 0.682 .8090313 1.382436

PA_Concern10 | .9799402 .0978858 -0.20 0.839 .8057001 1.191861

PA_Concern11 | .8577976 .1633027 -0.81 0.420 .5906604 1.245753

PA_Concern12 | .5885025 .1295542 -2.41 0.016 .3822637 .9060113

PA_Concern13 | 1.256963 .1805216 1.59 0.111 .9485833 1.665597

PA_Concern14 | 1.088364 .1260023 0.73 0.465 .8674188 1.365588

DSS_Experience1 | .7541846 .1703483 -1.25 0.212 .4844134 1.174192

DSS_Experience2 | 1.536328 .3716437 1.78 0.076 .9562575 2.468271

DSS_Experience3 | .8861134 .1657088 -0.65 0.518 .6141992 1.278408

DSS_Experience4 | .9419954 .1752715 -0.32 0.748 .6541402 1.356522

DSS_Experience5 | 1.170536 .2334297 0.79 0.430 .7918399 1.730344

DSS_Experience6 | 1.22933 .2375601 1.07 0.285 .8417424 1.795385

DSS_Experience7 | .827627 .1584641 -0.99 0.323 .5686654 1.204516

DSS_Experience8 | 1.357382 .2529227 1.64 0.101 .9420996 1.955724

DSS_Experience9 | 1.014932 .2125715 0.07 0.944 .6732215 1.530087

FarmSize_Acres | 1.000009 3.74e-06 2.35 0.019 1.000001 1.000016

Experience_Years | .988345 .0141772 -0.82 0.414 .9609451 1.016526

AgeGroup | .9489681 .1162807 -0.43 0.669 .7463639 1.20657

Female | 1.22235 .4046859 0.61 0.544 .6388319 2.338862

AdvancedDegree | 1.039259 .2362725 0.17 0.865 .6655886 1.622714

White | 1.271446 .3549149 0.86 0.390 .7356836 2.197377

SelfEmployed | 1.764809 .550807 1.82 0.069 .9572662 3.253588

EmployedAgEstablishment | 1.604034 .7066998 1.07 0.283 .6763855 3.803935

EmployedAgriBusiness | 1.353479 .3682441 1.11 0.266 .7940783 2.306959

_cons | .2383663 .3365909 -1.02 0.310 .0149722 3.794937

------------------------+----------------------------------------------------------------

AI_DSS2 |

AI_Perception1 | .9275936 .1452461 -0.48 0.631 .6824541 1.260788

AI_Perception2 | .9139736 .1422291 -0.58 0.563 .6737098 1.239922

AI_Perception3 | 1.076832 .1976773 0.40 0.687 .7514334 1.543141

AI_Perception4 | 1.47542 .2543972 2.26 0.024 1.052322 2.068631

AI_Perception5 | .6688106 .093479 -2.88 0.004 .5085475 .8795789

AI_Perception6 | 1.421853 .2151618 2.33 0.020 1.056932 1.91277

PA_Concern1 | 1.06859 .1858177 0.38 0.703 .7599677 1.502543

PA_Concern2 | 1.023486 .1677255 0.14 0.887 .7423178 1.411153

PA_Concern3 | .7029946 .1235281 -2.01 0.045 .4981744 .992025

PA_Concern4 | 1.229546 .1549258 1.64 0.101 .9604865 1.573977

PA_Concern5 | 1.13361 .1507937 0.94 0.346 .8734458 1.471265

PA_Concern6 | 1.346799 .1664464 2.41 0.016 1.057075 1.715932

PA_Concern7 | .6504387 .1084894 -2.58 0.010 .4690627 .9019486

PA_Concern8 | .8151027 .1796026 -0.93 0.353 .5292436 1.255362

PA_Concern9 | 1.383532 .2356457 1.91 0.057 .9908574 1.931824

PA_Concern10 | .8215822 .0991152 -1.63 0.103 .6485785 1.040733

PA_Concern11 | .8757971 .1907999 -0.61 0.543 .5714285 1.342286

PA_Concern12 | .7724461 .2165134 -0.92 0.357 .4459437 1.338001

PA_Concern13 | 1.109563 .1902109 0.61 0.544 .7929239 1.552645

PA_Concern14 | 1.166661 .1775363 1.01 0.311 .8657911 1.572085

DSS_Experience1 | .8604532 .2411308 -0.54 0.592 .4968086 1.490272

DSS_Experience2 | 1.767195 .4997798 2.01 0.044 1.015211 3.076185

DSS_Experience3 | .6765358 .1497515 -1.77 0.077 .4384064 1.04401

DSS_Experience4 | 1.10241 .2875497 0.37 0.709 .6611774 1.838096

DSS_Experience5 | 1.330686 .338972 1.12 0.262 .8076909 2.192332

DSS_Experience6 | 1.493932 .3603617 1.66 0.096 .9311216 2.396928

DSS_Experience7 | 1.020228 .2625851 0.08 0.938 .6160496 1.689579

DSS_Experience8 | 1.373676 .3176596 1.37 0.170 .873063 2.161338

DSS_Experience9 | .9102512 .2279688 -0.38 0.707 .5571616 1.487104

FarmSize_Acres | .999999 6.29e-06 -0.17 0.869 .9999866 1.000011

Experience_Years | .9791834 .0180763 -1.14 0.254 .9443879 1.015261

AgeGroup | 1.078011 .1614315 0.50 0.616 .8038151 1.44574

Female | 1.35515 .5730264 0.72 0.472 .5916384 3.103979

AdvancedDegree | .8010411 .2333114 -0.76 0.446 .45262 1.417672

White | 1.413365 .5350684 0.91 0.361 .67299 2.968248

SelfEmployed | 1.27387 .5400802 0.57 0.568 .5549359 2.924202

EmployedAgEstablishment | 1.541614 .8163846 0.82 0.414 .5460211 4.352533

EmployedAgriBusiness | 1.812116 .6768985 1.59 0.111 .8714206 3.768289

_cons | .0201836 .03783 -2.08 0.037 .0005124 .7950649

------------------------+----------------------------------------------------------------

AI_DSS3 |

AI_Perception1 | 1.097765 .2559065 0.40 0.689 .6951535 1.733557

AI_Perception2 | .9800479 .2264878 -0.09 0.931 .6230684 1.541554

AI_Perception3 | .950241 .2328622 -0.21 0.835 .5878165 1.536122

AI_Perception4 | 1.463392 .3818341 1.46 0.144 .8775292 2.440391

AI_Perception5 | .5239082 .1182174 -2.86 0.004 .3366554 .8153139

AI_Perception6 | 1.903608 .4344484 2.82 0.005 1.217064 2.977432

PA_Concern1 | 1.256448 .3273985 0.88 0.381 .7539508 2.093853

PA_Concern2 | 1.077927 .2865247 0.28 0.778 .6402223 1.81488

PA_Concern3 | .8210255 .1900718 -0.85 0.394 .5215536 1.292452

PA_Concern4 | 1.230874 .2285463 1.12 0.263 .8553901 1.771182

PA_Concern5 | .9497517 .1941048 -0.25 0.801 .6362773 1.417665

PA_Concern6 | 1.560407 .2623336 2.65 0.008 1.122367 2.169405

PA_Concern7 | .5952279 .1271559 -2.43 0.015 .3916019 .9047357

PA_Concern8 | .8325683 .2353241 -0.65 0.517 .4784419 1.448807

PA_Concern9 | 1.032218 .2680738 0.12 0.903 .6204532 1.717252

PA_Concern10 | .8030764 .1274897 -1.38 0.167 .5883385 1.096192

PA_Concern11 | 1.117033 .3214904 0.38 0.701 .635454 1.963578

PA_Concern12 | .9756141 .3447109 -0.07 0.944 .4881187 1.949982

PA_Concern13 | 1.07094 .2182058 0.34 0.737 .7183424 1.59661

PA_Concern14 | .7962037 .176068 -1.03 0.303 .5161718 1.228158

DSS_Experience1 | .7553499 .2919449 -0.73 0.468 .3541248 1.611165

DSS_Experience2 | 2.403697 1.023858 2.06 0.040 1.04306 5.539241

DSS_Experience3 | .9445686 .313725 -0.17 0.864 .492628 1.811123

DSS_Experience4 | .6378631 .2235609 -1.28 0.200 .3209186 1.267827

DSS_Experience5 | .9485368 .2936552 -0.17 0.864 .5170503 1.740106

DSS_Experience6 | 1.134709 .3673583 0.39 0.696 .6016044 2.140219

DSS_Experience7 | .8610958 .3063587 -0.42 0.674 .4287587 1.729378

DSS_Experience8 | 1.376706 .4324244 1.02 0.309 .7438378 2.548029

DSS_Experience9 | 1.969726 .6999839 1.91 0.056 .9815519 3.952741

FarmSize_Acres | .9999985 8.00e-06 -0.19 0.848 .9999828 1.000014

Experience_Years | .9771546 .0271692 -0.83 0.406 .925329 1.031883

AgeGroup | 1.12766 .2739924 0.49 0.621 .7004191 1.815508

Female | .9675143 .5810112 -0.05 0.956 .2981909 3.139211

AdvancedDegree | .4787274 .2113297 -1.67 0.095 .201527 1.137217

White | .717107 .3258674 -0.73 0.464 .294294 1.747377

SelfEmployed | 1.385479 .772263 0.58 0.559 .464667 4.131025

EmployedAgEstablishment | 2.18571 1.740356 0.98 0.326 .4590175 10.40772

EmployedAgriBusiness | 2.249897 1.260973 1.45 0.148 .7500667 6.748783

_cons | .0108024 .0306384 -1.60 0.110 .0000416 2.803848

-----------------------------------------------------------------------------------------

Note: Exponentiated coefficients represent odds ratios for alternative-specific variables (first equation) and relative-risk ratios for case-specific variables.

Note: _cons estimates baseline relative risk for each outcome.

.

. margins

Predictive margins Number of obs = 9,380

Model VCE: Robust

Expression: Pr(ChoiceSet), predict()

------------------------------------------------------------------------------

| Delta-method

| Margin std. err. z P>|z| [95% conf. interval]

-------------+----------------------------------------------------------------

_outcome |

Opt Out | .2554833 .0138327 18.47 0.000 .2283717 .2825949

AI-DSS1 | .4536801 .0155183 29.24 0.000 .4232649 .4840953

AI-DSS2 | .1923349 .0126606 15.19 0.000 .1675206 .2171492

AI-DSS3 | .0985017 .0109555 8.99 0.000 .0770294 .1199741

------------------------------------------------------------------------------
